# Prospective association between dietary pesticide exposure profiles and type 2 diabetes risk in the NutriNet-Santé cohort

**DOI:** 10.1186/s12940-022-00862-y

**Published:** 2022-05-25

**Authors:** Pauline Rebouillat, Rodolphe Vidal, Jean-Pierre Cravedi, Bruno Taupier-Letage, Laurent Debrauwer, Laurence Gamet-Payrastre, Hervé Guillou, Mathilde Touvier, Léopold K. Fezeu, Serge Hercberg, Denis Lairon, Julia Baudry, Emmanuelle Kesse-Guyot

**Affiliations:** 1 Nutritional Epidemiology Research Team (EREN), Epidemiology and Statistics Research Center, Sorbonne Paris Nord University, Inserm, INRAE, Cnam, University Paris Cité (CRESS), 74 rue Marcel Cachin, 93017 Bobigny, France; 2 Institut de L’Agriculture Et de L’Alimentation Biologiques (ITAB), 75595 Paris, France; 3 Toxalim (Research Centre in Food Toxicology), Université de Toulouse, INRAE, ENVT, INP-Purpan, UPS, 31027 Toulouse, France; 4 Département de Santé Publique, Hôpital Avicenne, 93017 Bobigny, France; 5 Aix Marseille Université, INSERM, INRAE, C2VN, 13005 Marseille, France

**Keywords:** Dietary exposure, Pesticides, Organic farming, Epidemiology, Type 2 diabetes, Environmental health

## Abstract

**Background:**

Studies focusing on dietary pesticides in population-based samples are scarce and little is known about potential mixture effects. We aimed to assess associations between dietary pesticide exposure profiles and Type 2 Diabetes (T2D) among NutriNet-Santé cohort participants.

**Methods:**

Participants completed a Food Frequency Questionnaire at baseline, assessing conventional and organic food consumption. Exposures to 25 active substances used in European Union pesticides were estimated using the *Chemisches und Veterinäruntersuchungsamt* Stuttgart residue database accounting for farming practices. T2D were identified through several sources.

Exposure profiles were established using Non-Negative Matrix Factorization (NMF), adapted for sparse data. Cox models adjusted for known confounders were used to estimate hazard ratios (HR) and 95% confidence interval (95% CI), for the associations between four NMF components, divided into quintiles (Q) and T2D risk.

**Results:**

The sample comprised 33,013 participants aged 53 years old on average, including 76% of women. During follow-up (median: 5.95 years), 340 incident T2D cases were diagnosed.

Positive associations were detected between NMF component 1 (reflecting highest exposure to several synthetic pesticides) and T2D risk on the whole sample: HR_Q5vsQ1_ = 1.47, 95% CI (1.00, 2.18). NMF Component 3 (reflecting low exposure to several synthetic pesticides) was associated with a decrease in T2D risk, among those with high dietary quality only (high adherence to French dietary guidelines, including high plant foods consumption): HR_Q5vsQ1_ = 0.31, 95% CI (0.10, 0.94).

**Conclusions:**

These findings suggest a role of dietary pesticide exposure in T2D risk, with different effects depending on which types of pesticide mixture participants are exposed to. These associations need to be confirmed in other types of studies and settings, and could have important implications for developing prevention strategies (regulation, dietary guidelines).

**Trial Registration:**

This study is registered in ClinicalTrials.gov (NCT03335644).

**Supplementary Information:**

The online version contains supplementary material available at 10.1186/s12940-022-00862-y.

## Introduction

Type 2 Diabetes (T2D) is a chronic disease characterized by chronic hyperglycaemia, resulting from inefficient use of insulin by the organisms’ cells. The number of people with diabetes around the world has increased from 108 million in 1980 (4.7%) to 451 million in 2017 (8.5%) [1, 2]. Furthermore, the number of deaths due to diabetes has increased by 70% worldwide between 2000 and 2019 [3]. A healthy diet – low in saturated fat, sweet products and rich in fiber – regular physical activity, maintaining a normal weight and avoiding smoking are known strategies to prevent or delay the onset of T2D [4] but environmental exposure, for instance food contaminants such as pesticides, may also play a role [5, 6]. 

Previous research has largely focused on organochlorine pesticides (OC) in relation with diabetes, mainly in occupational settings [7–10]. Several studies have provided information on the associations between increased T2D risk and OC exposure; for instance DDE, heptachlor, HCB, DDT, chlordane, with odds ratios ranging from 1.47 to 1.95 [9, 11]. A large proportion of these OCs have now been banned in the European Union legislation and replaced by other types of pesticides like organophosphorus (OP), neonicotinoid, and pyrethroid pesticides. Up to now, the latter have been far less investigated in relation with diabetes [12–14], especially through the dietary route, even though there are several mechanisms supporting potential effects [5, 15].

Moreover, in recent years, some studies have shown links between diabetes risk and organic food purchase or consumption [16], and recently a study conducted in the NutriNet-Santé cohort indicated inverse associations between a higher proportion of organic food in the diet and T2D risk [17]. These relationships could be explained by potentially lower concentrations of pesticides residues in plant organic foods, as organic agriculture regulation allows for a markedly smaller list of pesticides [18]. Consistently, organic food consumers exhibit lower urinary pesticide concentration than the non-organic consumers [19–22].

Moreover, it seems important when studying dietary exposure to pesticides, to consider exposure to mixtures, and not to compounds taken separately, as classically done in risk assessment studies.

In that context, the purpose of this work was to study the associations between dietary pesticide exposure profiles and T2D risk in a large sample of the NutriNet-Santé cohort.

## Material and methods

### Study population

The NutriNet-Santé study is a web-based prospective cohort of adults launched in France in May 2009 [23]. Self-administered validated questionnaires [24–29] were completed online at baseline by participants and administered every year. Dietary behaviors and specific health issues were collected through complementary questionnaires during follow-up.

### Dietary intake assessment

Between June and December 2014, the participants were invited to complete a 264-item web-based self-administered semi-quantitative food frequency questionnaire (Org-FFQ) differentiating organic and conventional foods. The Org-FFQ has been extensively described in other publications [30]. It was constructed on the basis of an existing validated FFQ [31] to which a 5-point ordinal scale was added to measure the frequency of organic food consumption. Participants provided the frequency of consumption and the quantity consumed for each item, assisted by photographs showing different portion sizes [32]. For food and beverages with an existing organic version (labelled), participants answered the question “*How often was the product of organic origin*?” by selecting 1 of the 5 following frequency modalities: never, rarely, half-of-time, often, or always. The organic food consumption was then calculated by matching to the modalities with respective percentage of, 0, 25, 50, 75, and 100. Weighting and sensitivity analyses for the Org-FFQ have been published elsewhere [30].

All food and beverage items were combined into 33 food groups. Nutritional values were obtained from a published food composition database [33]. A global proportion (as weight) of organic food in the diet was calculated as well as the proportion of organic food for each food group.

### Pesticide exposure assessment

Dietary exposure to pesticides was calculated by combining dietary intakes of each adult with pesticide residue concentration values in plant foods using contamination data from the European Union reference laboratory for pesticides, Chemisches und Veterinäruntersuchungsamt (CVUA) Stuttgart [34]. Contamination data for conventional and organic food products were both available in the database, details can be found in Supplementary Material 1. Among compounds available in this database, 25 commonly used pesticides were chosen, given both their frequency of detection above the Maximum Residue Levels (MRL) when sufficient data were available, and their frequency above Acceptable Daily Intake (ADI) otherwise, as detailed in Baudry et al. 2019 [35]. Pesticides authorized and widely used in organic farming processes (e.g. natural pyrethrins, spinosad) were also included. The 264 Org-FFQ items were broken down into 442 ingredients (comprising at least 5% of at least one food item). Animal-based ingredients were excluded, as CVUA encompassed plant-based ingredients only. Indeed, plant-based foods have notably more frequent and higher pesticides residues levels than foods of animal origin [36]. The resulting 180 plant ingredients were linked to the CVUA database and then were assigned a contamination value in organic and conventional farming modes (as the mean of corresponding data point). A description of the different steps for the decomposition and matching process is shown in Supplementary Material 2. The final 180 ingredients are listed in Supplementary Material 3.

For each ingredient/pesticide pair in conventional and organic farming, frequency of detection and frequency of quantification were determined using the formula as follows:

$$Frequency\;of\;detection=100\times\frac{Number\;of\;analyses-Number\;of\;undetected}{Number\;of\;analyses}$$

$$Frequency\;of\;quantification=100\times\frac{Number\;of\;analyses-Number\;of\;unquantified}{Number\;of\;analyses}$$

These frequencies were then used to determine the censoring rate in order to apply the most adapted methodology, following EFSA and WHO’s recommendations[37, 38].

Treatment of data below detection limit has been extensively described elsewhere [35, 39].

As food consumption data from NutriNet-Santé referred to edible foods (bone-free, peeled or cooked products), edibility and cooking factors were allocated to each ingredient as appropriate [40, 41]. Equal conversion factors were applied for both conventional and organic products. Cooking or peeling effects on pesticide residue levels were not considered as dilution factors were not known for all food/pesticide combinations [42]. The estimated daily intake (EDI) (in μg/kg of weight/day) under lower bound scenario was calculated for each of the selected pesticide and for each participant, following this formula:

$$\mathrm{EDI}={\textstyle\sum_{k=1}^{n\_i}}{\mathrm E}_{i,j}=({\mathrm C}_{i,k}\times{\mathrm L}_{k,j})/{\mathrm{Bw}}_i$$

E_i,j_ estimated daily exposure to pesticide j for individual i (µg/kg bw/day).

n_i number of plant foods in the diet of individual i.

C_i,k_ mean daily intake of plant food k by individual i (g/day).

L_k,j_ concentration of pesticide j in food k (mg/kg).

Bw_i_ body weight of individual i (kg).

Lower-bound (optimistic) rather than upper-bound scenario was used for this work, as upper-bound is known to overestimate exposure levels [35, 38, 43].

### Covariates

Baseline and yearly questionnaires collected sociodemographic and lifestyle characteristics such as gender, date of birth, occupation, educational level, and smoking practices. Monthly income by household unit was derived using both the household income and composition. Anthropometric measures (height, weight), physical activity (using the validated Physical Activity Questionnaire [44]) and family history of diabetes were also collected [24, 29]. Antihypertensive, lipid-lowering medications and self-declaration of hypertension or dyslipidemia were indicated at baseline through the health questionnaire. A multi-source approach was used to validate cardiovascular diseases [45].

**Fig. 1:**
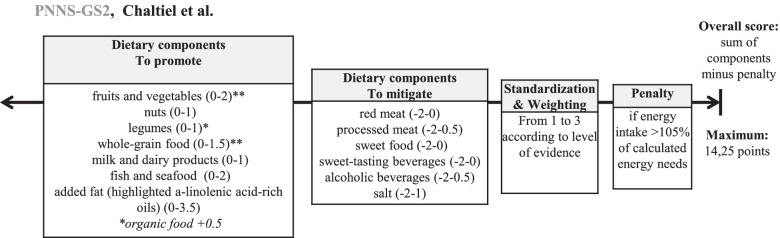
Scoring system for sPNNS-GS2 score

Concerning dietary data, the simplified Programme National Nutrition Santé Guidelines Score 2 (sPNNS-GS2), indicating the level of adherence to 2017 French dietary guidelines proposed by the High Council of Public Health [46, 47] and the provegetarian score [48] were computed for adjustment. The sPNNS-GS2 includes 13 components. Component, cut-off, scoring system and ponderation are presented in Fig. 1 and Supplementary Material 4.

The provegetarian score was computed as follows [48]: 7 plant food and 5 animal food groups were defined and sex-specific quintiles adjusted for total energy intake were calculated. For each plant component, 1 to 5 points were allocated to quintile 1 to 5 and for animal food groups the scoring was reversed. The provegetarian score was obtained by summing each quintile value of vegetable food group and each reverse quintile value of animal food group thus ranging from 12 (low plant food consumption) to 60 (high plant food consumption).

Three scores assessing the quality of the diet were also computed: Comprehensive Diet Quality Index (cDQI), plant-based Diet Quality Index (pDQI) and animal-based Diet Quality Index (aDQI) [49].

### Case ascertainment

Participants declared health events through a yearly health status questionnaire and a dedicated web-service at any time of the study. All medical records were compiled and examined by dedicated physicians. Physicians of participants declaring major health events were contacted to collect additional information if necessary. A medical expert committee validated these major health events.

Moreover, declared health data were merged with medico-administrative registers of the national health insurance system (Système National d’Information Inter-Régimes de l’Assurance Maladie [SNIIRAM] databases) to validate and provide information on health events. Mortality data from the French Centre for Epidemiology Medical Causes of Death database (CépiDC) were also used. Therefore, diabetes cases were identified using a multi-source approach, i.e. T2D self-reported during follow-up along with declaration of the use of T2D medication. Matching with the medico-administrative databases of the French National health insurance (SNIIRAM database) allowed us to correct potential errors. In this study, we considered as cases all T2D cases diagnosed between baseline (i.e. the date of completion of the Org-FFQ in 2014) and October 6^th^ 2020. Prevalent cases of type 1 diabetes and T2D were removed from the analysis.

### Statistical analyses

**Fig. 2:**
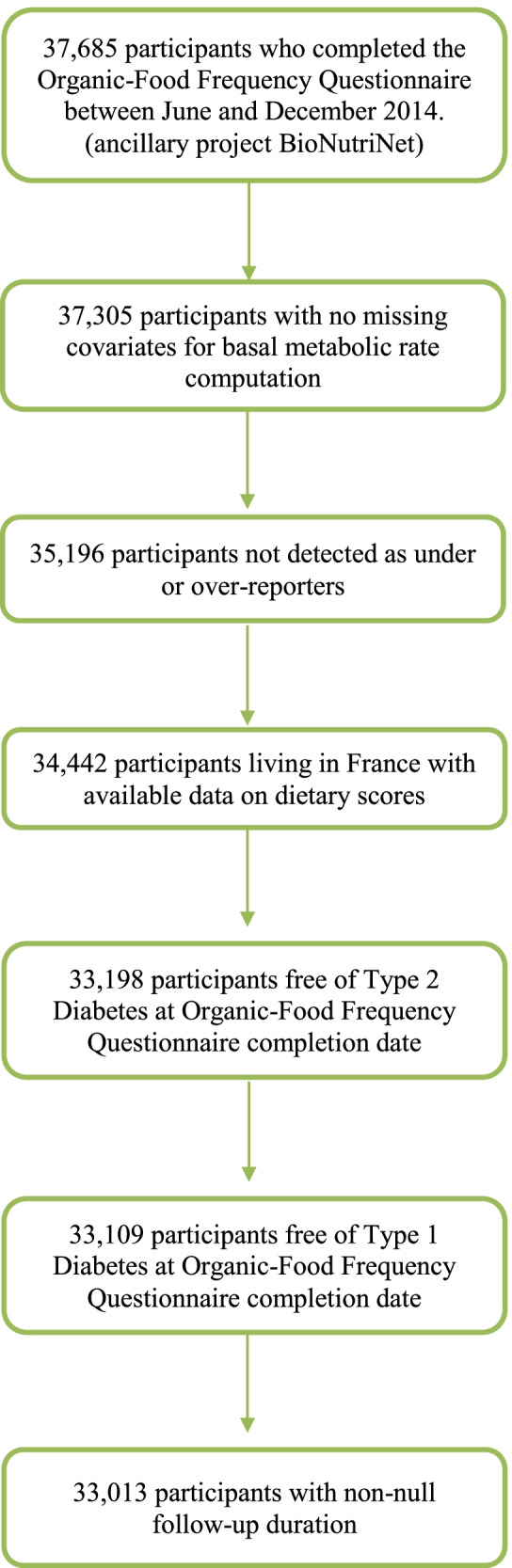
Flowchart for the sample selection, NutriNet-Santé Study, France, 2014 (*N* = 33,013)

A flowchart for the study sample selection is presented in Fig. 2.

For the present study, participants who completed the Org-FFQ between June and December 2014 (*N* = 37,685), with no missing covariates for basal metabolic rate computation (*N* = 37,305), who were not detected as under- or over-reporters (*N* = 35,196), living in France with available data on dietary scores (*N* = 34,442), who were free of type 1 diabetes or T2D when they completed the Org-FFQ (*N* = 33,013), were selected.

The detection method for under and over-reporters was based on the comparison between energy intake and energy requirement as previously performed in other studies [30, 50].

Dietary pesticide exposure profiles were analyzed using Non-Negative Matrix factorization (NMF) (detailed in Supplemental Material 5), specially adapted for non-negative data with excess zeros, developed by Lee et al. [51]. This method is frequently used in studies to identify mixtures of contaminants in the diet [52–55]. NMF procedure was performed using 25 selected pesticide exposure values (reflecting various pesticide exposure patterns) and resulted in four NMF components.

Chi^2^, Mantel-Haenzel, Wilcoxon and Kruskal–Wallis tests were applied as appropriate to compare sociodemographic and lifestyle characteristics, between cases and non-cases, and also across NMF-extracted component quintiles.

Cox proportional hazards regression models were used to assess associations between dietary pesticide exposure as profiles and T2D. NMF component scores were split into quintiles and introduced into separate models, with age as time scale, and first quintile as reference. Participants contributed person time until the date of diagnosis of diabetes, the date of last completed questionnaire, the date of death, or October 6^th^ 2020, whichever occurred first.

Cox models were adjusted for known potential confounders including smoking status, educational level, occupation, household income, physical activity, weight, height, family history of diabetes, overall quality of the diet (measured by the PNNS-GS2 score [47]). Interactions between gender and sPNNS-GS2 (overall nutritional quality of the diet), i.e. potential modulating factors, and NMF components were tested by introducing a multiplicative interaction term into the models. Interactions with *p* < 0.20 were further investigated by stratified analyses.

Schoenfeld residuals were used to test the proportional hazard assumption of the Cox model.

Potential nonlinear effects of continuous exposure variables were evaluated using martingale residuals. Tests for linear trend were performed using quintiles of the NMF components as ordinal variables.

Sensitivity analyses were performed to test for consistency of findings. A model excluding early cases (within 1 year after baseline), a model excluding CVD events before or during follow-up, and a model excluding baseline metabolic abnormalities (hypertension or dyslipidaemia) were performed as sensitivity analyses. Additional models were adjusted for provegetarian score and three scores assessing quality of foods, cDQI, pDQI and aDQI (data not shown) [49]. Two-sided tests were used. Data management and statistical analyses were performed using SAS (version 9.4; SAS Institute, Inc.). The NMF analysis was performed using R’s NMF package [56].

## Results

The baseline characteristics of the participants are presented in Table 1.

**Table 1:** Characteristics of the participants at baseline, Nutrinet-Santé Study, France, 2014 (*N* = 33,013)

	**All participants**	**Non-cases**	**T2D Cases**	***P*-value**^**a**^
**N**	33,013	32,673	340	
**Women**, %	76.36	76.57	56.76	< 0.0001
**Age**, mean (SD)	52.93 (13.99)	52.85 (14.01)	60.02 (9.39)	< 0.0001^b^
**Monthly income per household unit, %**				0.92
< €1200	11.58	11.58	12.35	
€1200–1800	22.99	22.98	24.41	
€1800–2700	27.22	27.24	25.29	
> €2700	31.94	31.95	31.76	
Unwilling to answer	6.26	6.27	6.18	
**Educational level**, %				< 0.0001
Less than high-school diploma	20.37	20.22	34.41	
High school diploma	14.6	14.56	18.53	
Post Graduate	65.03	65.22	47.06	
**Occupational status**, %				< 0.0001
Employee, manual worker	14.74	14.77	11.76	
Intermediate profession	15.32	15.36	10.59	
Managerial staff, intellectual	21.62	21.69	15.59	
Retired	35.08	34.92	50.88	
Self-employed, farmer	1.90	1.90	1.47	
Unemployed or never employed	11.34	11.35	9.71	
**Marital status, %**				< 0.0001
Single	12.78	12.81	9.71	
Divorced or separated	9.65	9.64	10.29	
Cohabiting	18.28	18.35	11.76	
Married	56.06	56.00	61.76	
Widowed	3.23	3.20	6.47	
**Physical activity**, %				0.03
High	33.27	33.29	30.88	
Moderate	36.79	36.82	34.12	
Low	19.19	19.12	25.59	
Missing data	10.75	10.77	9.41	
**Place of residence**, %				0.78
Rural community	22.36	22.36	22.65	
Urban unit with a population < 20,000 inhabitants	15.35	15.33	17.06	
Urban unit with a population between 20,000 and 200,000 inhabitants	18.51	18.51	18.82	
Urban unit with a population > 200,000 inhabitants	43.78	43.81	41.47	
**Smoking habits**, %				< .0001
Current smoker	11.07	11.07	11.47	
Former smoker	39.70	39.55	54.41	
Never smoker	49.22	49.38	34.12	
**Body Mass Index**, %				< .0001
< 25 kg/m^2^	67.31	67.82	18.23	
≥ 25 kg/m^2^ and < 30 kg/m^2^	24.06	23.90	39.71	
≥ 30 kg/m^2^	8.63	8.28	42.06	
**Family history of diabetes, %**	20.87	20.64	43.24	< .0001
**Cardiovascular disease at baseline or during follow-up, %**	0.73	0.72	1.47	0.10
**Hypertriglyceridaemia at baseline, %**	2.71	2.58	15.29	< .0001
**Hypercholesterolemia at baseline, %**	16.73	16.47	41.76	< .0001
**Hypertension at baseline, %**	10.88	10.62	36.47	< .0001

^a^*p*-value for Chi-square tests comparing cases and non-cases unless otherwise stated

^b^*p*-value for Wilcoxon test comparing cases and non-cases

The sample, aged on average of 53 years old (SD 14) at baseline, was constituted of 76% of women. Monthly income of 1800€-2700€ was the most represented category. Sixty-five percent of the participants had a post-graduate diploma, and more than 50% were married. Most represented place of residence was urban units with more than 200,000 inhabitants.

Participants who developed a T2D during follow up (340 cases) were more frequently men and older subjects. Differences between cases and non-cases were observed for educational level, with less post-graduated participants in cases as well as occupation, with more retired cases compared to non-cases. Cases were more often married and more likely to be former smokers.

Participants with a BMI above 25 kg/m^2^ represented 82% of cases compared to 33% for non-cases. Family history of diabetes was more frequent in cases.

Considering nutritional parameters (Table 2), cases had higher energy and alcohol intakes. Organic proportion in the diet was 16% (SD 0.18) in cases, compared to 22% (SD 0.21) in non-cases (unadjusted descriptive figures). Non-cases more often followed a vegetarian or vegan diet. For information, sociodemographic and nutritional characteristics, compared across quintiles of each NMF component can be found in Supplementary Tables 1 to 8.

**Table 2:** Nutritional characteristics of the participants, NutriNet-Santé Study, France, 2014 (*N* = 33,013)

	**All participants**	**Non-cases**	**Cases**	*P*-value^a^
**N**	33,013	32,673	340	
**Energy intake without Alcohol,** kcal/day	1936 (612)	1934 (611)	2119 (690)	< 0.0001
**Ethanol**, grams/day	8.36 (12.24)	8.33 (12.20)	11.33 (15.41)	0.001
**sPNNS-GS2 score**	2.78 (3.41)	2.79 (3.41)	1.25 (3.59)	< 0.0001
**provegetarian score**	36.06 (5.85)	36.08 (5.85)	34.05 (5.58)	< 0.0001
**Special diet,** %				0.02^b^
Omnivorous	95.37	95.34	98.82	
Pesco-Vegetarian	1.83	1.84	0.59	
Vegetarian	1.88	1.89	0.29	
Vegan	0.93	0.93	0.29	
**PANDiet score** (/100)	65.02 (7.90)	65.04 (7.90)	62.95 (7.21)	< 0.0001
**Carbohydrates** (% of alcohol-free energy intake)	39.72 (7.52)	39.73 (7.52)	38.36 (7.19)	0.0005
**Lipids** (% of alcohol-free energy intake)	41.18 (7.12)	41.17 (7.12)	41.38 (6.57)	0.60
**Protein** (% of alcohol-free energy intake)	18.71 (3.69)	18.70 (3.68)	19.88 (3.72)	< 0.0001
Plant / total protein ratio	0.34 (0.15)	0.34 (0.15)	0.29 (0.11)	< 0.0001
**Proportion of individuals with organic food in the diet ≥ 50%**	0.13 (0.33)	0.13 (0.33)	0.07 (0.26)	0.0039
**Proportion of organic food in the diet**	0.22 (0.21)	0.22 (0.21)	0.16 (0.18)	< 0.0001

*sPNNS-GS2:* simplified Programme National Nutrition Santé Score 2

*PANDiet*: Diet Quality Index Based on the Probability of Adequate Nutrient Intake

^a^*p*-value for Student or Wilcoxon tests comparing cases and non-cases as appropriate

^b^*p*-value for Chi-square test comparing cases and non-cases

Regarding pesticide exposure, correlations between selected pesticides and NMF Components are shown in Table 3. NMF Component 1 was highly correlated (coefficients > 0.60) with azoxystrobin, chlorpyriphos, imazalil, malathion, profenofos and thiabendazole. High positive correlations with NMF Component 2 were observed for azoxystrobin, boscalid, cyprodinil, difenoconazole, fenhexamid, iprodione, lambda-cyhalothrin and tebuconazole. NMF Component 3 was characterized by high correlations with spinosad. High correlations for NMF Component 4 were found for acetamiprid, carbendazim, chlorpyrifos, cypermethrin, and dimethoate/omethoate. Among the active substances that are listed, some of them are no longer authorized in the EU in plant protection products as indicated in Table 3. For information, absolute values for the estimated pesticide exposure in μg/kg of weight/day are presented in Supplementary Tables 9 and 10.

**Table 3:** Spearman Correlations between 25 selected pesticides and NMF Components, NutriNet-Santé Study, 2014 (*N* = 33,013)

	**Spearman Correlation Coefficients**			
	**NMF** **Component 1**	**NMF** **Component 2**	**NMF** **Component 3**	**NMF** **Component 4**
**Acetamiprid**	0.35	0.42	0.30	**0.85**
**Anthraquinone**^**a**^	0.16	0.16	-0.05	0.19
**Azadirachtin**	-0.07	0.03	0.55	0.04
**Azoxystrobin**	**0.61**	**0.70**	-0.14	0.15
**Boscalid**	0.51	**0.90**	-0.06	0.19
**Carbendazim**^**a**^	0.32	0.38	0.36	**0.88**
**Chlorpropham**^**a**^	0.33	0.52	-0.30	0.07
**Chlorpyrifos**^**a**^	**0.72**	0.43	0.17	**0.61**
**Cypermethrin**	0.31	0.28	0.41	**0.92**
**Cyprodinil**	0.50	**0.90**	-0.06	0.17
**Difenoconazole**	0.53	**0.68**	0.07	0.47
**Dimethoate/Omethoate**^**a**^	0.37	0.43	0.31	**0.78**
**Fenhexamid**	0.47	**0.79**	-0.07	0.12
**Glyphosate**	0.37	0.45	-0.10	0.15
**Imazalil**	**1.00**	0.36	-0.09	0.16
**Imidacloprid**^**a**^	0.53	0.16	0.18	0.53
**Iprodione**^**a**^	0.52	**0.90**	-0.04	0.16
**Lambda Cyhalothrin**	0.56	**0.83**	-0.01	0.26
**Malathion**^**a**^	**0.73**	0.49	-0.07	0.18
**Methamidophos**^**a**^	0.29	0.31	-0.21	0.15
**Profenofos**^**a**^	**0.94**	0.36	-0.11	0.19
**Pyrethrins**	0.06	0.02	0.19	0.04
**Spinosad**	-0.06	-0.02	**0.99**	0.38
**Tebuconazole**	0.56	**0.83**	-0.05	0.20
**Thiabendazole**	**0.99**	0.34	-0.10	0.17

Bold values denote correlation coefficients > 0.60

*NMF* Non-negative Matrix Factorization

^a^these active substances are no longer authorized in the European Union

Table 4 presents spearman correlations between food consumption and NMF Components: NMF Component 1 was particularly positively correlated with conventional fruit and fruit juice intakes. NMF Component 2 was positively correlated with conventional fruits and negatively with several organic food groups (potatoes, vegetables or legumes). NMF Component 3 was positively correlated with plant-based organic food groups (soup, vegetables, fruits, potatoes) while NMF Component 4 exhibited positive correlations with non-alcoholic drinks and weak correlations with organic food groups.

**Table 4:** Spearman Correlations between dietary intakes for 33 food groups and NMF components (continuous), NutriNet-Santé Study, 2014, *N* = 33,013

NMF Components	Conventional food groups				Organic food groups			
	**1**	**2**	**3**	**4**	**1**	**2**	**3**	**4**
Alcohol	0.01	0.08	0.02	-0.04	-0.19	**-0.22**	**0.33**	-0.02
Bread	0.10	0.08	-0.16	-0.03	-0.11	-0.13	0.18	-0.01
Butter	0.07	0.09	-0.05	-0.02	-0.19	**-0.20**	**0.30**	-0.04
Cereals	0.03	0.03	-0.03	0.04	**-0.23**	**-0.24**	**0.37**	0.01
Cheese	0.05	0.09			**-0.21**	**-0.24**	**0.35**	-0.03
Cookies	0.05	-0.01	-0.10	0.01	**-0.20**	**-0.23**	**0.28**	-0.02
Dairy products	0.19	0.19	-0.02	0.08	-0.13	-0.14	**0.29**	0.01
Dressing	0.09	0.10	-0.03		**-0.23**	**-0.27**	**0.37**	-0.05
Eggs	0.05	0.10	0.09		-0.13	-0.13	**0.34**	
Fast food	0.04		-0.08	0.03	**-0.23**	**-0.29**	**0.36**	-0.04
Fat	0.03	0.03	0.04	0.04	**-0.23**	**-0.23**	**0.36**	-0.02
Fish	0.12	**0.20**	0.10	0.06	-0.14	-0.17	0.30	-0.01
Fruit juice	**0.36**	-0.10	0.02	0.15	-0.01	**-0.23**	**0.28**	0.07
Fruits	**0.35**	**0.44**	**0.23**	0.04	-0.19	**-0.21**	**0.53**	-0.03
Grains	-0.09	-0.07	0.25	0.06	-0.14	-0.12	0.29	0.05
Legumes	-0.02	0.02	0.15	0.01	**-0.23**	**-0.25**	0.40	-0.03
Meat	0.13	0.18	-0.13	-0.07	-0.14	-0.19	**0.32**	-0.05
Milk	0.06		-0.11	-0.05	-0.03	-0.08	0.05	-0.02
Milk soy	-0.11	-0.10	0.16	0.04	-0.15	-0.14	0.20	0.02
Milky desserts	0.07	0.02	-0.09	0.01	-0.13	-0.16	0.22	-0.02
Non-alcoholic drinks	0.03	0.07	**0.28**	**0.36**	**-0.23**	**-0.23**	**0.41**	0.10
Nuts	0.03	0.06	**0.23**	0.12	-0.18	-0.17	**0.37**	0.03
Oil		0.11	**0.26**	0.04	-0.24	**-0.22**	**0.43**	-0.03
Potatoes	0.04	0.14	-0.04	-0.11	-0.25	**-0.34**	**0.46**	-0.08
Poultry	0.10	0.13	-0.09	0.02	-0.13	-0.16	**0.29**	-0.03
Processed meat	0.09	0.10	-0.13	-0.03	-0.18	**-0.21**	**0.31**	-0.04
Red meat	0.12	0.17	-0.12	-0.07	-0.15	-0.19	**0.31**	-0.06
Snacks		0.02	0.07	0.03	**-0.21**	**-0.23**	**0.35**	
Soda	0.08	-0.01	-0.14	0.02	-0.11	-0.16	0.15	
Soup	0.10	0.16	**0.21**	0.08	-0.19	**-0.26**	**0.50**	-0.01
Soy	-0.16	-0.14	**0.20**	0.04	-0.18	-0.16	**0.22**	0.03
Sweetened food	0.09	0.09	0.02	0.01	**-0.22**	**-0.27**	**0.44**	-0.03
Vegetables	0.08	0.32	0.40	0.03	**-0.27**	**-0.31**	**0.68**	-0.06
White meat	0.12	0.15	-0.14	-0.03	-0.16	-0.19	**0.33**	-0.04
Whole products	-0.07	-0.05	**0.21**	0.06	**-0.21**	**-0.21**	**0.35**	
Water	0.05	0.05						

Non-significant correlations are not presented (blank cells) / *NMF* Non-negative Matrix Factorization / Bold values denote correlation coefficients ≥ 0.20 or ≤ -0.20

Pesticide residue data was available only for plant-based products

Cox models’ results for associations between dietary pesticide exposure and T2D risk are shown in Table 5. Median follow-up time was 5.95 years. In the extensively adjusted model (model 3), an increased risk of T2D was found for quintiles 3, 4 and 5 of NMF Component 1: HR_Q5 vs Q1_ = 1.47 (95% CI = 1.00, 2.18), p-trend 0.048. No significant associations were found for NMF Component 2 with HR_Q5vsQ1_ = 1.11, 95% CI (0.76, 1.62), nor NMF Component 4, HR_Q5vsQ1_ = 0.80, 95% CI (0.54, 1.18), nor Component 3, HR_Q5vsQ1_ = 0.88, 95% CI (0.60, 1.29). A model adjusted for fruit and vegetable intake (instead of sPNNS-GS2 score) was also performed and showed very similar results although somewhat attenuated (data not shown).

**Table 5:** Cox models for associations between dietary pesticide exposure and Type 2 diabetes, NutriNet-Santé Study, France, 2014 (*N* = 33,013)

	**Quintile 1**	**Quintile 2**	**Quintile 3**	**Quintile 4**	**Quintile 5**	**Total**	***P*-value for trend**
**Number of participants**	6602	6603	6603	6603	6602	33,013	
**NMF Component 1**							
Incident Cases	52	83	77	72	56	340	
Person-years	33,331.09	34,305.64	34,129.91	34,229.60	33,908.04	169,904.28	
Model 1, HR (95% CI)	1	1.59 (1.13, 2.25)	1.48 (1.04, 2.10)	1.32 (0.92, 1.88)	1.05 (0.72, 1.54)		0.74
Model 2, HR (95% CI)	1	1.37 (0.96, 1.94)	1.51 (1.06, 2.15)	1.44 (1.00, 2.07)	1.40 (0.95, 2.06)		0.10
Model 3, HR (95% CI)	1	1.36 (0.96, 1.93)	1.53 (1.07, 2.18)	1.48 (1.03, 2.12)	1.47 (1.00, 2.18)		0.048
**NMF Component 2**							
Incident Cases	59	70	73	73	65	340	
Person-years	33,476.37	33,815.55	34,159.62	34,349.49	34,103.25	169,904.28	
Model 1, HR (95% CI)	1	1.13 (0.80, 1.60)	1.07 (0.76, 1.51)	1.00 (0.71, 1.41)	0.88 (0.62, 1.26)		0.33
Model 2, HR (95% CI)	1	1.07 (0.75, 1.51)	1.10 (0.78, 1.57)	1.14 (0.80, 1.63)	1.06 (0.73, 1.56)		0.66
Model 3, HR (95% CI)	1	1.01 (0.71, 1.43)	1.07 (0.75, 1.52)	1.12 (0.79, 1.61)	1.11 (0.76, 1.62)		0.46
**NMF Component 3**							
Incident Cases	95	76	67	59	43	340	
Person-years	33,492.56	33,875.27	34,051.61	34,183.11	34,301.73	169,904.28	
Model 1, HR (95% CI)	1	0.90 (0.61, 1.33)	0.83 (0.61, 1.12)	0.66 (0.49, 0.88)	0.45 (0.31, 0.64)		< .0001
Model 2, HR (95% CI)	1	0.89 (0.60, 1.31)	1.01 (0.75, 1.37)	0.94 (0.69, 1.27)	0.79 (0.54, 1.16)		0.36
Model 3, HR (95% CI)	1	0.95 (0.70, 1.29)	1.06 (0.77, 1.46)	1.00 (0.71, 1.41)	0.88 (0.60, 1.29)		0.73
**NMF Component 4**							
Incident Cases	103	76	70	53	38	340	
Person-years	33,655.47	33,739.32	33,877.51	34,142.43	34,489.55	169,904.28	
Model 1, HR (95% CI)	1	0.89 (0.60, 1.31)	0.83 (0.62, 1.12)	0.79 (0.58, 1.08)	0.52 (0.38, 0.73)		< .0001
Model 2, HR (95% CI)	1	1.02 (0.69, 1.51)	0.99 (0.73, 1.34)	1.01 (0.73, 1.38)	0.79 (0.57, 1.11)		0.16
Model 3, HR (95% CI)	1	1.02 (0.76, 1.38)	1.13 (0.83, 1.54)	0.86 (0.61, 1.21)	0.80 (0.54, 1.18)		0.24

*NMF* Non-Negative Matrix Factorization, *HR* Hazard Ratio, *95% CI* 95% Confidence Interval. // Median follow-up = 5.95 years

Model 1 adjusted for age (time-scale), gender. / Model 2 adjusted for age (time-scale), gender, physical activity (IPAQ), smoking status, educational level, occupation, monthly income per household unit, marital status, alcohol-free energy intake, family history of diabetes, weight, height. / Model 3 adjusted for model 2 + sPNNS-GS2 score

Stratifications were performed when p for interaction was < 0.20: *p* = 0.03 for interaction between NMF Component 1 and gender, *p* = 0.08 for sPNNS-GS2 tertiles and NMF Component 2, *p* = 0.15 for sPNNS-GS2 tertiles and NMF Component 3.

After stratification by gender (Table 6), the association remained only in women: HR_Q5vsQ1_ = 1.28 (95% CI = 1.00, 2.84), p-trend 0.003.

**Table 6:** Stratified analyses of the association of dietary pesticide exposure (NMF Components) and risk of Type 2 Diabetes, NutriNet-Santé Study, France, 2014 (*N* = 33,013)

	**Quintile 1**	**Quintile 2**	**Quintile 3**	**Quintile 4**	**Quintile 5**	**Number of individuals**	***P*-value for trend**	**P for interaction**
**Gender**^a^								
NMF Component 1, Women	1	1.09 (0.66, 1.80)	1.54 (0.95, 2.50)	1.98 (1.24, 3.16)	1.69 (1.00, 2.84)	25,210	0.003	0.03
NMF Component 1, Men	1	1.79 (1.07, 3.00)	1.45 (0.83, 2.52)	1.12 (0.62, 2.01)	1.28 (0.70, 2.33)	7803	0.87	
**sPNNS-GS2 Score**^b^								
NMF Component 2, Tertile 1	1	0.83 (0.52, 1.33)	0.91 (0.57, 1.46)	0.94 (0.58, 1.52)	0.86 (0.51, 1.44)	11,004	0.75	0.08
NMF Component 2, Tertile 2	1	0.97 (0.50, 1.86)	0.85 (0.43, 1.67)	0.95 (0.49, 1.86)	1.28 (0.65, 2.52)	11,098	0.56	
NMF Component 2, Tertile 3	1	1.70 (0.67, 4.30)	2.76 (1.14, 6.70)	3.40 (1.36, 8.46)	2.38 (0.90,6.27)	10,911	0.03	
NMF Component 3, Tertile 1	1	1.00 (0.63, 1.57)	1.18 (0.76,1.82)	0.99 (0.60,1.62)	0.99 (0.60, 1.66)	11,004	0.95	0.15
NMF Component 3, Tertile 2	1	1.40 (0.79, 2.49)	0.92 (0.47, 1.81)	1.19 (0.63, 2.27)	0.93 (0.44, 1.93)	11,098	0.74	
NMF Component 3, Tertile 3	1	0.79 (0.41,1.51)	0.84 (0.42, 1.69)	0.83 (0.41, 1.71)	0.31 (0.10, 0.94)	10,911	0.10	

^a^Models adjusted for age (time-scale), physical activity (IPAQ), smoking status, educational level, monthly income per household unit, marital status, alcohol-free energy intake, family history of diabetes, weight, height, sPNNS-GS2 score

^b^Models adjusted for age (time-scale), gender, physical activity (IPAQ), smoking status, educational level, monthly income per household unit, marital status, alcohol-free energy intake, family history of diabetes, weight, height, sPNNS-GS2 score

When stratifying by sPNNS-GS2 score, positive associations were found for quintiles 3–4 of NMF Component 2, in sPNNS-GS2 tertile 3 (highest adherence to French dietary guidelines). A negative association was evidenced in the same tertile for quintile 5 of NMF Component 3, HR_Q5vsQ1_ = 0.31 (95% CI = 0.10, 0.94).

Several sensitivity analyses were performed. Additional adjustments for provegetarian score, pDQI, cDQI or aDQI did not change results (data not shown).

After excluding T2D cases within 1 year after baseline (Table 7), similar trends were observed but association was no longer significant for NMF Component 1, HR_Q5vsQ1_ = 1.50 (95% CI = 0.99, 2.27). Similar magnitude was found when excluding CVD cases before or during follow-up, for NMF Component 1, HR_Q5vsQ1_ = 1.47, 95% CI (0.99, 2.18), p-trend 0.04.

**Table 7:** Sensitivity analyses for associations between dietary pesticide exposure and Type 2 Diabetes risk, NutriNet-Santé Study, France, 2014 (*N* = 33,013)

	**Quintile 1**	**Quintile 2**	**Quintile 3**	**Quintile 4**	**Quintile 5**	***P*-value for trend**
**Excluding Early T2D Cases** (within 1 year of baseline), *N* = 32,967, 294 T2D cases						
NMF Component 1	1	1.35 (0.93, 1.97)	1.35 (0.92, 1.99)	1.44 (0.98, 2.12)	1.50 (0.99, 2.27)	0.06
NMF Component 2	1	1.05 (0.72, 1.52)	0.99 (0.68, 1.45)	1.03 (0.70, 1.52)	1.19 (0.79, 1.77)	0.49
NMF Component 3	1	1.06 (0.70, 1.60)	1.18 (0.85, 1.64)	1.01 (0.73, 1.41)	0.90 (0.59, 1.37)	0.78
NMF Component 4	1	1.09 (0.71, 1.66)	1.10 (0.79, 1.53)	1.16 (0.83, 1.63)	0.83 (0.58, 1.20)	0.51
**Excluding CVD**^**a**^** cases before or during follow-up**, *N* = 32,773, 235 T2D cases						
NMF Component 1	1	1.34 (0.94, 1.91)	1.55 (1.09, 2.22)	1.50 (1.04, 2.16)	1.47 (0.99, 2.18)	0.04
NMF Component 2	1	0.97 (0.68, 1.39)	1.06 (0.75, 1.51)	1.15 (0.80, 1.65)	1.11 (0.76, 1.63)	0.39
NMF Component 3	1	0.91 (0.61, 1.35)	1.07 (0.79, 1.46)	1.02 (0.75, 1.38)	0.87 (0.59, 1.29)	0.79
NMF Component 4	1	1.04 (0.70, 1.54)	0.99 (0.73, 1.35)	1.06 (0.77, 1.45)	0.83 (0.59, 1.16)	0.27
**Excluding metabolic abnormalities**^**b**^** at baseline**, *N* = 24,962, 129 T2D cases						
NMF Component 1	1	1.41 (0.75, 2.64)	2.17 (1.19, 3.95)	1.82 (0.98, 3.39)	1.81 (0.93, 3.52)	0.05
NMF Component 2	1	0.65 (0.36, 1.18)	0.87 (0.50, 1.54)	1.12 (0.65, 1.94)	1.17 (0.66, 2.07)	0.22
NMF Component 3	1	1.18 (0.65, 2.14)	0.95 (0.56, 1.61)	0.97 (0.58, 1.63)	1.11 (0.61, 2.01)	0.86
NMF Component 4	1	1.23 (0.66, 2.30)	1.57 (0.95, 2.59)	1.14 (0.66, 1.98)	1.20 (0.69, 2.07)	0.74

All models were adjusted for age (time-scale), gender, physical activity (IPAQ), smoking status, educational level, monthly income per household unit, marital status, alcohol-free energy intake, family history of diabetes, weight, height, sPNNS-GS2 score

^a^Participants developing a cardiovascular disease during the follow-up or before were excluded from the sample

^b^Participants with hypertension or dyslipidaemia at baseline were removed

Finally, excluding metabolic abnormalities at baseline did not substantially change the results for NMF Component 1, but statistical power seemed reduced: HR_Q5vsQ1_ = 1.81, 95% CI (0.93, 3.52), p-trend 0.05.

## Discussion

In this large sample of French adults, we observed positive associations between NMF component 1 (reflecting higher exposure to a synthetic pesticides mixture of azoxystrobin, chlorpyriphos, imazalil, malathion, profenofos, thiabendazole) and T2D risk. After stratification on gender, these associations remained only in women. Further analysis revealed a T2D risk increase in association with NMF Component 2 and a T2D risk decrease in association with NMF Component 3, but only in the third tertile of sPNNS-GS2 score (high adherence to French dietary guidelines).

To our knowledge, the present work is the first to evaluate the associations between dietary pesticide exposure profiles and T2D risk in a large population-based sample. As a result, our findings cannot be directly compared to prior scientific literature.

However, some studies have been conducted to investigate associations between occupational, residential or domestic pesticide exposure and T2D risks [9].

Whilst research has been carried out on OCs [57, 58], now banned in the European Union, there is still little evidence on OPs, pyrethroids and neonicotinoids. However, associations between environmental exposure to pyrethroids and increased risk of all-cause and cardiovascular disease mortality and increased risk of T2D were recently found in two studies conducted in the US National Health and Nutrition Examination Survey [59, 60].

A study among wives of pesticides applicators of the Agricultural Health Study, conducted in Iowa and North Carolina (United States), did observe associations between some OPs and increased diabetes risk. However, these OPs (fonofos, phorate and parathion) were not included in our selected list [12]. Interestingly, another study among pesticide applicators of the Agricultural Health Study found a positive dose–response association between cumulative use of chlorpyrifos and incident diabetes [13]. In addition, a study in male farmers evidenced a positive correlation between malathion blood concentration, waist circumference and insulin resistance [14].

Despite different magnitudes due to the differences in exposure pathways, these results are consistent with our study where we found an association between NMF Component 1, positively correlated with malathion and chlorpyriphos, and incident T2D.

The fact that this association remains only in women after stratification on gender, could be linked to differential detoxification processes in men and women [61] or to limited power due to a low proportion of men in the cohort.

The negative associations between NMF Component 3 and T2D risk found in our study may partly be explained by the fact that this component is also negatively correlated with a few synthetic pesticides (azoxystrobin, chlorpropham, methamidophos) as well as being highly correlated with some pesticides used in organic farming (i.e. natural pyrethrins, spinosad). In addition to being less exposed to the synthetic studied pesticides, participants with high NMF Component 3 score also seemed particularly less exposed to pesticides with highly suspected toxicity such as chlorpyriphos, imazalil and malathion. The association was detected in the third tertile of sPNNS-GS2 score only (highest adherence to French dietary guidelines), where the participants have the highest consumptions of fruits and vegetables and also highest proportions of organic food in their diet. These results reflect those of a study by Kesse-Guyot et al. in 2020, in the same cohort, who also reported a negative association between high organic food score and T2D risk (HR_Q5_ = 0.65; 95% CI(0.43; 0.97)) [62]. It is possible, as stated in this study, that organic farming regulations lead to a lower frequency or an absence of synthetic pesticide residues in organic foods compared with conventional foods thus conferring lower T2D risk. Furthermore, the observed effects had corresponding magnitude in the two studies.

Pesticides are biologically active compounds and their mechanisms of action and cellular targets are similar to those involved in the development of metabolic syndrome and hepatic complications in mammals [63]. Therefore, they can be considered as metabolic disrupting contaminants able to influence T2D development. Mechanisms underpinning these associations could be linked to the impact of pesticide alone or to mixture on glucose and lipid metabolisms [64]. Indeed, some insecticides like imidacloprid, can stimulate cholinergic receptors, which can lead to disorders of insulin and glucagon secretions [65]. Pesticides could also affect other insulin sensitive tissues such as liver [63]. In addition, pesticides can disturb intra-cellular mechanisms in the adipose tissue, leading to excessive adipogenesis and overweight or obesity, which is an important risk factor for diabetes [66, 67]. Finally, numerous pesticides are now considered as certain or possible endocrine disruptors, able to alter estrogen or androgen actions. These effects can then lead to obesity and diabetes [68, 69].

### Limitations and strengths

Some limitations of this study should be noted. Firstly, the NutriNet-Santé cohort consists of volunteers, with a higher education level and higher incomes, who are possibly more preoccupied by their health and dietary intakes than the general French population [70, 71]. Therefore, caution is needed when extrapolating our results to other populations. These characteristics can also have consequences on health outcomes with lower prevalence and incidence of diseases, than those observed in the general population. In a previous study on T2D conducted in the NutriNet-Santé cohort, T2D incidence was found lower: 186 cases per 100 000 person-years in the sample after standardization vs 289 per 100,000 in the French population [72, 73].

Secondly, this is an observational study, therefore the causality of the observed associations cannot be established and residual confounding cannot be entirely ruled out.

It is possible that risk alpha inflated with multiple comparisons. However, our analyses were hypothesis-driven, supported by available data in animal or mechanistic studies in the literature and the number of outcomes and subgroup analyses were limited.

Other limitations, inherent to the pesticide database, could be mentioned: for instance, data were not available for animal products (with generally low levels of pesticides) and the database did not contain measures for copper or sulfur-based products, widely used in organic farming. Measures were performed in Germany, but products from all over European Union were tested. Pesticide exposure was estimated based on dietary intakes, since the financial burden limits biomarker monitoring in very large cohorts. Some urinary metabolites measures were available for a limited sub-sample of the cohort (300 individuals). For information, the expected links between pesticides used in the present study and available urinary metabolites are presented in Supplementary Table 11. Means and standard deviations for urinary concentrations of several metabolites for the sub-sample, in each quintile of NMF Components are presented in Supplementary Table 12 and Supplementary Table 13. It would be very interesting to have complete biomarker data. However, this type of data would not allow to identify precisely compounds to which participants are exposed. Another source of uncertainty could origin from potential concentration or dilution effects during washing, cooking or peeling on pesticide residue levels that we were not able to account for [42].

Some strengths of this study can also be advanced. First of all, this work proposes to study several compounds at the same time, via the approach by NMF profiles, unlike classical studies in the field where molecules are evaluated separately which neglects potential synergistic effects whereas mixtures are present even at very low doses [15, 68, 69, 74].

Moreover, a wide range of covariates were taken into account for adjustment in the Cox regression models, including major potential confounders such as diet quality indicators and lifestyle factors. In spite of limited number of diabetes cases, the sample size still allowed to compute additional stratified and sensitivity models in order to improve comprehension of these results and reduce confounding bias.

## Conclusion

We observed a positive association between NMF Component 1, highly correlated to a mixture of synthetic pesticides such as azoxystrobin, chlorpyriphos, imazalil, malathion, profenofos, thiabendazole, and T2D risk. Another important finding was the negative association between lower synthetic pesticide exposure profile (through NMF component 3) and diabetes risk, specifically in those with a healthy diet. A positive association for NMF Component 2 was also found after stratification on French dietary guidelines only for those with a healthy diet (highest adherence to French dietary guidelines). Some published experimental studies provide basic knowledge explaining, at least partly, these observations.

These associations should be examined in other prospective studies, in diverse settings, to complement these observational studies in order to validate estimated dietary pesticide exposure. The pesticide mixtures found in this study could be administrated to animals in order to better understand underlying mechanisms. If confirmed by other studies, these findings may help to understand the role of dietary pesticide exposure in major chronic diseases’ incidence. These results would have important implications for developing prevention strategies for the whole population, through regulation or dietary guidelines. 

## Supplementary Information

**Additional file 1:** **Supplementary Material1. **Description of the *Chemischesund Veterinäruntersuchungsamt*pesticide exposure database. **Supplementary Material 2.** Flowchart fordecomposition of ingredients and matching. **Supplementary Material 3.** 180 ingredients afterdecomposition. **Supplementary Material 4.** Details on thecomputation of the simplified Programme National Nutrition Santé Guideline Score2 (sPNNS-GS2 score). **Supplementary Material 5.** Details of the Non-Negative Matrix Factorization (NMF) procedure. **Supplementary Table 1. **Characteristics of quintiles forNMF Component 1, NutriNet-Santé Study, 2014 (*N*=33,013). **Supplementary Table 2.** Nutritional characteristics of the participants across quintiles of NMFcomponent 1, NutriNet-Santé Study, 2014 (*N*=33,013). **Supplementary Table 3.** Characteristics of quintiles for NMF Component 2, NutriNet-Santé Study, 2014(*N*=33,013). **Supplementary Table 4 .** Nutritional characteristics of the participants across quintiles of NMFcomponent 2, NutriNet-Santé Study, 2014 (*N*=33,013). **Supplementary Table 5. **Characteristics of quintiles for NMF Component 3, NutriNet-Santé Study, 2014(*N*=33,013). **Supplementary Table 6 .** Nutritional characteristics of the participants across quintiles of NMFcomponent 3, NutriNet-Santé Study, 2014 (*N*=33,013). **Supplementary Table 7.** Characteristics of quintiles for NMF Component 4, NutriNet-Santé Study, 2014(*N*=33,013). **Supplementary Table 8 . **Nutritional characteristics of the participants across quintiles of NMFcomponent 4, NutriNet-Santé Study, 2014 (*N*=33,013). **Supplementary Table 9.** Estimated dietary pesticide exposure across NMF Components 1 and 2 quintiles(in μg/kg of weight/day), NutriNet-Santé Study, 2014 (*N*=33,013). **Supplementary Table 10.** Estimated dietary pesticide exposure across NMF Components 3 and 4 quintiles(in μg/kg of weight/day), NutriNet-Santé Study, 2014 (*N*=33,013). **Supplementary Table 11.** Expected links between metabolites and parent compounds. **Supplementary Table 12.** Urinaryconcentrations (µg/g creatinine) for parent compounds and metabolites in NMFComponents 1 and 2 quintiles, NutriNet-Santé Study (*N*=296). **Supplementary Table 13.** Urinaryconcentrations (µg/g creatinine) for parent compounds and metabolites in NMFComponents 3 and 4 quintiles, NutriNet-Santé Study (*N*=296). 

## Data Availability

Researchers from public institutions can submit a collaboration request including information on the institution and a brief description of the project to collaboration@etude-nutrinet-sante.fr. All requests will be reviewed by the steering committee of the NutriNet-Santé study. A financial contribution may be requested. If the collaboration is accepted, a data access agreement will be necessary and appropriate authorizations from the competent administrative authorities may be needed. In accordance with existing regulations, no personal data will be accessible.

## Abbreviations

ADI: Acceptable Daily Intake
BMI: Body Mass Index
CépiDC: French Centre for Epidemiology Medical Causes of Death database
CI: Confidence Interval
CNIL: Commission Nationale de l’Informatique et des Libertés
CVUA: Chemisches und Veterinäruntersuchungsamt
DDE: Dichlorodiphenyldichloroethylene
DDT: Dichlorodiphenyltrichloroethane
EDI: Estimated Daily Intake
EFSA: European Food and Safety Authority
FFQ: Food Frequency Questionnaire
HCB: Hexachlorobenzene
HR: Hazard Ratio
IPAQ: International Physical Activity Questionnaire
IRB INSERM: Institutional Review Board of the French Institute for Health and Medical Research
NMF: Non-negative Matrix Factorization
OC: Organochlorine
OP: Organophosphorous
PNNS: Programme National Nutrition Santé
Q: Quintile
SD: Standard Deviation
SNIIRAM: Système National d’Information Inter-Régimes de l’Assurance Maladie
T2D: Type 2 diabetes
WHO: World Health Organization
