# Supplementary material for: Prospective association between dietary pesticide exposure profiles and type 2 diabetes risk in the NutriNet-Santé cohort
Source: Environ Health. 2022 May 25;21:57. doi: 10.1186/s12940-022-00862-y (PMC9131692; doi:10.1186/s12940-022-00862-y)
Supplement: Supplementary file 1 — Additional file 1: Supplementary Material1. Description of the Chemischesund Veterinäruntersuchungsamtpesticide exposure database. Supplementary Material 2. Flowchart fordecomposition of ingredients and matching. Supplementary Material 3. 180 ingredients afterdecomposition. Supplementary Material 4. Details on thecomputation of the simplified Programme National Nutrition Santé Guideline Score2 (sPNNS-GS2 score). Supplementary Material 5. Details of the Non-Negative Matrix Factorization (NMF) procedure. Supplementary Table 1. Characteristics of quintiles forNMF Component 1, NutriNet-Santé Study, 2014 (N=33,013). Supplementary Table 2. Nutritional characteristics of the participants across quintiles of NMFcomponent 1, NutriNet-Santé Study, 2014 (N=33,013). Supplementary Table 3. Characteristics of quintiles for NMF Component 2, NutriNet-Santé Study, 2014(N=33,013). Supplementary Table 4 . Nutritional characteristics of the participants across quintiles of NMFcomponent 2, NutriNet-Santé Study, 2014 (N=33,013). Supplementary Table 5. Characteristics of quintiles for NMF Component 3, NutriNet-Santé Study, 2014(N=33,013). Supplementary Table 6 . Nutritional characteristics of the participants across quintiles of NMFcomponent 3, NutriNet-Santé Study, 2014 (N=33,013). Supplementary Table 7. Characteristics of quintiles for NMF Component 4, NutriNet-Santé Study, 2014(N=33,013). Supplementary Table 8 . Nutritional characteristics of the participants across quintiles of NMFcomponent 4, NutriNet-Santé Study, 2014 (N=33,013). Supplementary Table 9. Estimated dietary pesticide exposure across NMF Components 1 and 2 quintiles(in μg/kg of weight/day), NutriNet-Santé Study, 2014 (N=33,013). Supplementary Table 10. Estimated dietary pesticide exposure across NMF Components 3 and 4 quintiles(in μg/kg of weight/day), NutriNet-Santé Study, 2014 (N=33,013). Supplementary Table 11. Expected links between metabolites and parent compounds. Supplementary Table 12. Urinaryconcentrations (µg/g [file 12940_2022_862_MOESM1_ESM.docx]

**Supplementary Materials and Supplementary Tables**

Supplementary Material 1**:** Description of the *Chemisches und Veterinäruntersuchungsamt* pesticide exposure database.
Supplementary Material 2: Flowchart for decomposition of ingredients and matching
Supplementary Material 3: 180 ingredients after decomposition

Supplementary Material 4: Details on the computation of the simplified Programme National Nutrition Santé Guideline Score 2 (sPNNS-GS2 score)
Supplementary Material 5: Details of the Non-Negative Matrix Factorization (NMF) procedure

[**Supplementary Table 1:** Characteristics of quintiles for NMF Component 1, NutriNet-Santé Study, 2014 (N=33,013) 8](#_Toc102064117)

[Supplementary Table 2: Nutritional characteristics of the participants across quintiles of NMF component 1, NutriNet-Santé Study, 2014 (N=33,013) 9](#_Toc102064118)

[Supplementary Table 3: Characteristics of quintiles for NMF Component 2, NutriNet-Santé Study, 2014 (N=33,013) 10](#_Toc102064119)

[Supplementary Table 4 : Nutritional characteristics of the participants across quintiles of NMF component 2, NutriNet-Santé Study, 2014 (N=33,013) 11](#_Toc102064120)

[Supplementary Table 5: Characteristics of quintiles for NMF Component 3, NutriNet-Santé Study, 2014 (N=33,013) 12](#_Toc102064121)

[Supplementary Table 6 : Nutritional characteristics of the participants across quintiles of NMF component 3, NutriNet-Santé Study, 2014 (N=33,013) 13](#_Toc102064122)

[Supplementary Table 7: Characteristics of quintiles for NMF Component 4, NutriNet-Santé Study, 2014 (N=33,013) 14](#_Toc102064123)

[Supplementary Table 8 : Nutritional characteristics of the participants across quintiles of NMF component 4, NutriNet-Santé Study, 2014 (N=33,013) 15](#_Toc102064124)

[Supplementary Table 9: Estimated dietary pesticide exposure across NMF Components 1 and 2 quintiles (in μg/kg of weight/day), NutriNet-Santé Study, 2014 (N=33,013) 16](#_Toc102064125)

[Supplementary Table 10: Estimated dietary pesticide exposure across NMF Components 3 and 4 quintiles (in μg/kg of weight/day), NutriNet-Santé Study, 2014 (N=33,013) 17](#_Toc102064126)

[Supplementary Table 11: Expected links between metabolites and parent compounds 18](#_Toc102064127)

[Supplementary Table 12: Urinary concentrations (µg/g creatinine) for parent compounds and metabolites in NMF Components 1 and 2 quintiles, NutriNet-Santé Study (N=296) 19](#_Toc102064128)

[Supplementary Table 13: Urinary concentrations (µg/g creatinine) for parent compounds and metabolites in NMF Components 3 and 4 quintiles, NutriNet-Santé Study (N=296) 20](#_Toc102064129)

**Supplementary Material 1:** Description of the *Chemisches und Veterinäruntersuchungsamt* pesticide exposure database.

The *Chemisches und Veterinäruntersuchungsamt Stuttgart* was designated in 2006 as the European Union Reference Laboratory for pesticide residues measures.

The database comprises contamination data for plant-based foods from 88 different countries (both inside and outside the European Union), available on the European Union market. It contains a wide range of pollutants such as pesticides, hormones, and other pollutants. Analyses over four years were aggregated (2012-2015) for this study, resulting in a database with more than 6 million datapoints (i.e., a result for a pesticide residue and a food product), including 1 million for organic food products.

The CVUAS database contains information on the commodity and compound names (in German and English), the production system (organic or conventional), the date of analysis, the country of origin, the unit of measurement, the limit of detection (LOD) and limit of quantification (LOQ), and three modalities for the result of the analysis: 'undetected,' 'unquantified,' or a numeric value.

The median number of analyses for conventional pesticide/food pairs was 23 and 6 for organic pesticide/food pairs.

Distribution of the number of analyses performed according to the method of production

| Production system | Minimum | P25 | P50 | P75 | Maximum |
| --- | --- | --- | --- | --- | --- |
| Conventional | 1 | 3 | 23 | 84 | 353 |
| Organic | 1 | 2 | 6 | 16 | 64 |

**Supplementary Material 2:** Flowchart for decomposition of ingredients and matching

264 items^a^

Decomposition of Org-FFQ items into ingredients

766 ingredients

Exclusion of ingredients with a

proportion <5% within an item

442 ingredients

Matching with the CVUA database (exclusion of animal-based ingredients)

180 ingredients^b^

**Abbreviations:** CVUA: *Chemisches und Veterinäruntersuchungsamt* / Org-FFQ: Organic Food Frequency Questionnaire. ^a^ : 264 Org-FFQ items : Avocado, artichoke, onion, garlic, mushroom, green salad, carrots, celery, tomato, beetroot, red cabbage, white cabbage, green cabbage, Brussels sprouts, cauliflower, broccoli, green beans, endive, spinach, cucumber, pepper, leek, fennel, pumpkin, turnip, peas, corn, seaweed, Vegetable soup, Fruit puree, Fruit puree without sugar, fruits in syrup, apple, pear, citrus, banana, peach, apricot, melon, cherry, strawberry, plum, kiwi, grape, pineapple, mango, lychee, exotic fruit, fruit juice, pure fruit juice, dried fruits, shellfish, crustaceans, breaded fish, fatty fish, fish, lean fish, rabbit, chopped steak, roasted beef, beef ribs, braised beef, "pot au feu", veal escalope, roasted veal, veal cutlet, veal sauté, lamb ribs, lamb shoulder, lamb sauté, pork ribs, roasted pork, pork loin, pork tenderloin, liver, tripe, breaded meat, ham, raw ham, dry sausage, saveloy, mortadella, "paté", "rillettes", bacon, sausages, Turkey, turkey with skin, egg "à la coque", fried egg, whole milk, half-skimmed milk, skimmed milk, fermented milk, whole yoghurt, whole fruit yoghurt, half-skimmed yoghurt, half-skimmed fruit yoghurt , yoghurt without fat, fruit yoghurt without fat, yoghurt without fat with aspartame, bifidus yoghurt , fruit bifidus yoghurt, white cheese without fat, white cheese without fat with aspartame, white cheese 20% fat, white cheese with fruit 20% fat, white cheese 40% fat, white cheese 40% fat (sheep), white cheese 40 % fat (goat), white cheese 40% fat with fruits, petit suisse, processed cheese, blue cheese, brie cheese, goat cheese, gouda cheese, edam cheese, mozzarella cheese, light cheese, fresh cheese, entremet, "crème caramel", potato salad, boiled potatoes, fried potatoes , potato puree, french fries, Jerusalem artichoke, White bread, rusk, plain breakfast cereals, muesli breakfast cereals, plain muesli, bran cereals, cereal cake, semolina, quinoa, white rice, pasta, whole grain pasta, brown rice, wild rice, whole grain bread, sunflower oil, olive oil, peanut oil, rapeseed oil, corn oil, soybean oil, mixed oil, walnut oil, hazelnut oil, seed oil, sesame oil, coconut oil, linseed oil, safflower oil, butter for cooking, butter for toast, viennoiseries, brioche, dry chocolate biscuit, dry biscuit, small cake, coffee with milk, coffee with plant milk, black coffee, chicoree with milk, chicoree with plant milk, chicoree , hot chocolate, hot chocolate with plant milk, tea, infusion, tea with milk, tea with milk and mineral water, spring water, tap water, alcohol-free beer, kombucha, juice nectar, syrup, soda, diet soda, honey and jam, oleaginous puree, nutella, sugar, sweetener, whipped cream, chocolate, candy, fruit tart, flan, cake, brownie, cream cake, chocolate bar, crêpe with sugar, sorbet, ice cream, cone, flavoured cereals, crêpe with salted garniture, quiche, croque-monsieur, pizza, ravioli, Greek sandwich, Asian food, hamburger, panini, sauerkraut, cassoulet, vegan ham, vegan chorizo, vegan pâté, soy steak, vegetarian patties, tofu, seitan, meat substitute, Mustard, ketchup, tartare sauce, béchamel sauce, pasta sauce, meat sauce, juice sauce, vinaigrette, Cider, beer, white wine, red wine, aniseed-flavoured alcohol, appetizer, strong alcohol, liqueur, digestif, cocktail, salted crackers, chips, popcorn, salted oleaginous, unsalted oleaginous, beer yeast, seeds, bran, wheat germ, sprouted seeds, Mayo, crème fraîche, light crème fraîche, vegetable cream, soy yoghurt, vegan fresh cheese, vegan soy cheese, vegan cheese without soy, vegan soy dessert, soy-free vegan dessert, soy milk, legumes.

^b^ : 180 ingredients detailed in **Supplementary Material 3**

**Supplementary Material** **3**: 180 ingredients after decomposition

apricot

cooked apricot

dried apricot

fresh garlic

unsalted roasted almonds

unsalted almonds

pineapple

pineapple, cooked

artichoke

artichoke, raw

eggplant

avocado

banana

batavia

beet

wheat (Ebly type)

chard

bulgur

broccoli

nectarine (“brugnon”)

unsalted roasted peanuts

coffee

decaffeinated coffee

espresso coffee

carrot

raw carrot

celery stalk

celery stalk raw

celery root raw

celery root, cooked

cherry

raw mushroom

button mushroom

dehydrated black mushrooms

raw white cabbage

cooked white cabbage

Brussels sprouts

raw red cabbage

cooked red cabbage

cooked green cabbage

sauerkraut

cauliflower

raw cauliflower

yellow lemon

clementine / mandarin

applesauce with no added sugar

cucumber

zucchini

dried date

shallot

raw endive

cooked endive

spinach

raw spinach

white flour

whole grain flour

corn flour

rice flour

buckwheat flour

rye flour

corn starch (Maïzena)

potato starch

fennel

cooked fennel

oak leaf salad

bean

dry fig

flageolet bean

oat flakes

artichoke hearts

strawberry

cooked strawberry

raspberry

passion fruit (maracudja)

wheat germ

sprouted seed

coriander seeds

pumpkin seeds

flax seeds

sesame seeds

sunflower seeds

pomegranate

white bean

red bean

green beans

peanut oil

rapeseed oil

flaxseed oil

corn oil

walnut oil

sesame oil

soybean oil

sunflower oil

olive oil

unspecified oil

pure pineapple juice

squeezed lemon juice

cranberry juice

freshly squeezed grapefruit juice

pure grapefruit juice

pure apple juice

pure grape juice

freshly squeezed orange juice

pure orange juice

pure orange juice with pulp

kiwi fruit

lettuce

soy lecithin

lentils

fresh lychee

lamb's lettuce

sweet corn

corn on the cob

mango

regular margarine

melon

mirabelle plum

nectarine

unsalted hazelnuts

nuts

unsalted cashew nuts

fresh coconut

raw onion (yellow, red, white)

cooked onion (yellow, red, white)

black olive

orange

grapefruit

papaya

sesame paste (tahini)

pasta

whole grain pasta

peach

cooked peach

peas

unsalted pistachios

pear

cooked pear

leek

raw yellow bell pepper

raw red bell pepper

raw green bell pepper

apple

baked potato

steamed potato

boiled potato

unsalted popcorn

pumpkin

soy protein

plum

prune

diced tomato pulp

almond puree

radish

black radish

white grapes

black grape

dried raisins

cooked rhubarb

white rice (polished)

brown rice

wild rice

green salad

mixed green salad

salsify

escarole salad

semolina (couscous seed)

cornmeal (polenta)

cooked soybeans

oat bran

wheat bran

tea

herbal tea, infusion

tomato

cherry tomato

cooked tomato

Jerusalem artichoke

white wine

rosé wine

red wine

**Supplementary Material 4:** Details on the computation of the simplified Programme National Nutrition Santé Guideline Score 2 (sPNNS-GS2 score)

| Dietary components | Recommendation | Criteria ^1^ | Score |
| --- | --- | --- | --- |
| Fruits and vegetables  (weight=3) | At least 5 servings/day, with 1 max as juice and 1 max as dried | [0 - 3.5[  [3.5 - 5[  [5 - 7.5[  ≥7.5 | 0  0.5  1  2 |
|  | Prefer organic fruits | Most of the time  Occasionally  Never | 0.5  0.25  0 |
|  | Prefer organic vegetables | Most of the time  Occasionally  Never | 0.5  0.25  0 |
| Nuts  (weight=1) | A handful/day | 0  ]0 – 0.5[  [0.5- 1.5[  ≥1.5 | 0  0.5  1  0 |
| Legumes  (weight=1) | At least 2 servings/week | 0 /week  ]0-2[ /week  ≥2 /week | 0  0.5  1 |
|  | Prefer organic legumes | Most of time  Occasionally  Never | 0.5  0.25  0 |
| Whole-grain food  (weight=2) | Every day | 0  ]0 - 1[  [1 - 2[  ≥2 | 0  0.5  1  1.5 |
|  | Prefer organic bread | Most of the time  Occasionally  Never | 0.5  0.25  0 |
|  | Prefer organic grains | Most of the time  Occasionally  Never | 0.5  0.25  0 |
| Milk and dairy products  (weight=1) | 2 servings/day | [0 - 0.5[  [0.5 - 1.5[  [1.5 - 2.5[  ≥2.5 | 0  0.5  1  0 |
| Red meat  (weight=2) | Limit consumption | [0 - 500[ g/week  [500 - 750[ g/week  ≥750 g/week | 0  -1  -2 |
| Processed meat  (weight=3) | Limit consumption | [0 - 150[ g/week  [150 - 300[ g/week  ≥300 g/week | 0  -1  -2 |
|  | Prefer white ham over other processed meat ^2^ | Ratio <50%  Ratio ≥50% | 0  0.5 |
| Fish and seafood  (weight=2) | 2 servings/week | [0 - 1.5[servings/week  [1.5 - 2.5[servings/week  [2.5 - 3.5[servings/week  ≥3.5 servings/week | 0  1  0.5  0 |
|  | Fatty fish 1 serving/week | [0 - 0.5[servings/week  [0.5 - 1.5[ servings/week  ≥1.5 servings/week | 0  1  0 |
| Added fat  (weight=2) | Avoid overeating | >16% of EIWA  ≤16% of EIWA | 0  1.5 |
|  | Prefer ALA-rich and olive oil over other oils | Ratio <50%  Ratio ≥50% | 0  1 |
|  | Prefer vegetal fat over animal fat | Ratio >50%  Ratio ≤50% | 0  1 |
| Sugary foods  (weight=3) | Limit consumption | <10 % of EIWA  [10-15[% of EIWA  ≥15% of EIWA | 0  -1  -2 |
| Sweet-tasting beverages ^3^  (weight=3) | Limit consumption | 0 mL/day  ]0 - 250[ mL/day  [250 - 750[ mL/day  ≥ 750mL mL/day | 0  -0.5  -1  -2 |
| Alcoholic beverages  (weight=3) | Limit consumption | 0 g/week  ]0-100] g/week  ]100-150]  ]150-200] g/week  >200 g/week | 0.5  0  -1  -1.5  -2 |
| Salt  (weight=3) | Limit consumption | <6 g/day  [6-8[ g/day  [8-10[g/day  [10-12[ g/day  ≥12 g/day | 1  0  -0.5  -1  -2 |

**Abbreviations:** EIWA: energy intake without alcohol, ALA: α-linolenic acid, PNNS-GS2: Programme
National Nutrition Santé – Guideline Score 2

^1^Servings per day unless otherwise is stated

^2^Conditional: the 0.5 bonus point only occurs if total processed meat consumption is more than 150 g/week

^3^Sweetened beverages are specifically sugary sweeten beverages, artificially sweetened beverages and fruit juices

**Supplementary Material 5:** Details of the Non-Negative Matrix Factorization (NMF) procedure

Non-negative Matrix Factorization (NMF) is a non-supervised data decomposition method, proposed by Lee^1^ to deal with non-negative data using non-negativity constraints.
This method is relevant for non-negative data with excess zeros and measurement error such as exposure to pesticides constrained by the detection limits of assay techniques.
The purpose of NMF is to explain observed data through a limited number of components approximating the original data as accurately as possible.
The matrix representing the basis components and the matrix of mixture coefficients are constrained to have non-negative values, and no orthogonality or independence constraints are imposed on the basis components.

Let X be a matrix (n × p) containing only non-negative values and without a row or column containing only 0 and r a relatively small integer < n and < p.

The non-negative factorization of matrix X is the search for two matrices W (n× r) and H (r ×p) containing only positive or zero values and whose product approaches X so that X ≈ WH.

The factorization is solved by searching for a local optimum of the optimization problem:

min W, H ≥ 0[ L (X , WH) ]

L is a loss function measuring approximation quality. Since the objective is usually to reduce the dimension of the original data, the factorization rank r is in practice often chosen such that r << min(n, p). This equation is solved by a multiplicative algorithm based on a gradient descent approach.

**Supplementary Table 1:** Characteristics of quintiles for NMF Component 1, NutriNet-Santé Study, 2014 (N=33,013)

| **NMF Component 1** | **Quintile 1** | **Quintile 2** | **Quintile 3** | **Quintile 4** | **Quintile 5** | **P-value**  **for trend** | **P^1^** |
| --- | --- | --- | --- | --- | --- | --- | --- |
| N | 6602 | 6603 | 6603 | 6603 | 6602 |  |  |
| **Woman**, % | 74.70 | 75.07 | 75.59 | 75.60 | 80.85 | <.0001 | <.0001 |
| **Age**, years, mean (SD) | 52.71  (13.65) | 51.68  (14.03) | 52.15  (14.00) | 53.56  (13.89) | 54.54  (14.18) | <.0001 | <.0001 |
| **Monthly income**  **per household unit**, % |  |  |  |  |  | 0.001 | <.0001 |
| <€1200 | 13.94 | 11.62 | 10.98 | 10.48 | 10.91 |  |  |
| €1200-1800 | 23.90 | 23.44 | 23.26 | 22.19 | 22.16 |  |  |
| €1800-2700 | 26.34 | 27.46 | 27.88 | 27.93 | 26.48 |  |  |
| >€2700 | 29.26 | 31.59 | 32.26 | 33.79 | 32.82 |  |  |
| Unwilling to answer | 6.56 | 5.89 | 5.62 | 5.62 | 7.63 |  |  |
| **Educational level**, % |  |  |  |  |  | 0.03 | 0.0002 |
| Less than high-school diploma | 21.07 | 19.70 | 18.99 | 20.40 | 21.68 |  |  |
| High school diploma | 14.65 | 13.66 | 14.51 | 15.27 | 14.93 |  |  |
| Post Graduate | 64.28 | 66.64 | 66.50 | 64.33 | 63.39 |  |  |
| **Occupational status**, % |  |  |  |  |  | 0.04 | <.0001 |
| Employee, manual worker | 15.24 | 15.31 | 14.84 | 14.04 | 14.28 |  |  |
| Intermediate profession | 14.31 | 15.51 | 16.51 | 15.90 | 14.34 |  |  |
| Managerial or intellectual  profession | 21.87 | 23.67 | 23.02 | 21.64 | 17.92 |  |  |
| Retired | 33.25 | 32.14 | 33.42 | 36.70 | 39.91 |  |  |
| Self-employed, farmer | 2.74 | 2.18 | 1.56 | 1.58 | 1.42 |  |  |
| Unemployed or never employed | 12.59 | 11.2 | 10.64 | 10.14 | 12.11 |  |  |
| **Marital status, %** |  |  |  |  |  |  |  |
| Single | 13.83 | 12.72 | 12.37 | 11.93 | 13.06 | 0.0002 | <.0001 |
| Divorced or separated | 9.91 | 9.13 | 8.75 | 10.07 | 10.39 |  |  |
| Cohabiting | 19.31 | 19.96 | 19.40 | 17.25 | 15.48 |  |  |
| Married | 53.97 | 55.40 | 56.32 | 57.40 | 57.19 |  |  |
| Widowed | 2.98 | 2.79 | 3.15 | 3.35 | 3.88 |  |  |
| **Place of residence**, % |  |  |  |  |  |  |  |
| Rural community | 24.19 | 23.96 | 22.57 | 21.40 | 19.69 | <.0001 | <.0001 |
| Urban unit with a population  <20,000 inhabitants | 15.92 | 15.67 | 15.39 | 15.43 | 14.31 |  |  |
| Urban unit with a population  between 20,000 and 200,000 | 18.81 | 17.93 | 18.25 | 18.58 | 18.98 |  |  |
| Urban unit with a population  >200,000 inhabitants | 41.08 | 42.44 | 43.80 | 44.59 | 47.02 |  |  |
| **Smoking habits,** % |  |  |  |  |  |  |  |
| Current smoker | 12.31 | 12.06 | 11.24 | 10.33 | 9.44 | <.0001 | <.0001 |
| Former smoker | 41.59 | 38.66 | 37.85 | 40.71 | 39.70 |  |  |
| Never smoker | 46.09 | 49.28 | 50.92 | 48.96 | 50.86 |  |  |
| **Body Mass Index >25 kg/m^2^**, % | 33.01 | 37.03 | 33.83 | 34.48 | 25.10 | <.0001 | <.0001 |
| **Physical activity**, % |  |  |  |  |  |  |  |
| High | 33.60 | 30.90 | 31.74 | 33.97 | 36.14 | 0.48 | <.0001 |
| Moderate | 35.07 | 38.86 | 37.71 | 36.32 | 35.99 |  |  |
| Low | 21.04 | 20.13 | 20.11 | 18.28 | 16.39 |  |  |
| Missing data | 10.30 | 10.12 | 10.43 | 11.43 | 11.48 |  |  |
| **Family history of diabetes**, % | 20.71 | 20.51 | 21.73 | 20.64 | 20.78 | 0.43 | 0.86 |

^1^P-value for Chi-square comparisons or linear regressions as appropriate across quintiles.
NMF: Non-negative Matrix Factorization

Supplementary Table 2: Nutritional characteristics of the participants across quintiles of NMF component 1, NutriNet-Santé Study, 2014 (N=33,013)

| **NMF Component 1** | **Quintile 1** | **Quintile2** | **Quintile 3** | **Quintile 4** | **Quintile 5** | **P-value  for trend** | **P-value^a^** |
| --- | --- | --- | --- | --- | --- | --- | --- |
| **N** | 6602 | 6603 | 6603 | 6603 | 6602 |  |  |
| **Energy intake without**  **Alcohol,** kcal/day | 1791 (600) | 1844 (578) | 1936 (598) | 2012 (606) | 2098 (626) | <.0001 | <.0001 |
| **Ethanol**, grams/day | 8.57 (13.37) | 8.40 (12.03) | 8.21 (11.43) | 8.69 (12.14) | 7.91 (12.15) | <.0001 | 0.002 |
| **sPNNS-GS2 score** | 3.21 (3.45) | 2.55 (3.40) | 2.58 (3.38) | 2.59 (3.40) | 2.94 (3.39) | <.0001 | <.0001 |
| **Provegetarian score** | 37.41 (6.39) | 35.48 (5.66) | 35.49 (5.66) | 35.75 (5.67) | 36.16 (5.62) | <.0001 | <.0001 |
|  |  |  |  |  |  |  |  |
| **Special diet,** % |  |  |  |  |  | <.0001 | <.0001 |
| Omnivorous | 90.81 | 96.32 | 96.35 | 97.06 | 96.32 |  |  |
| Pesco-Vegetarian | 3.32 | 1.42 | 1.58 | 1.33 | 1.48 |  |  |
| Vegetarian | 3.89 | 1.67 | 1.39 | 1.08 | 1.35 |  |  |
| Vegan | 1.98 | 0.59 | 0.68 | 0.53 | 0.85 |  |  |
|  |  |  |  |  |  |  |  |
| **PANDiet score** (/100) | 65.26 (8.26) | 64.31 (7.56) | 64.90 (7.61) | 65.11 (7.81) | 65.54 (8.18) | <.0001 | <.0001 |
| **Carbohydrates**  (% of alcohol free  energy intake) | 38.45 (8.19) | 38.81 (7.31) | 39.37 (7.04) | 40.28 (7.12) | 41.70 (7.42) | <.0001 | <.0001 |
| **Lipids** (% of alcohol-  free energy intake) | 42.92 (7.78) | 41.91 (6.87) | 41.29 (6.63) | 40.43 (6.68) | 39.33 (7.03) | <.0001 | <.0001 |
| **Protein** (% of alcohol-free energy intake) | 18.35 (4.20) | 18.96 (3.72) | 18.97 (3.48) | 18.85 (3.47) | 18.43 (3.47) | <.0001 | <.0001 |
| Plant /  total protein ratio | 0.39 (0.19) | 0.33 (0.14) | 0.32 (0.13) | 0.32 (0.13) | 0.33 (0.13) | <.0001 | <.0001 |
| **Proportion of individuals with organic food in the diet ≥ 50%** | 0.36 (0.48) | 0.11 (0.32) | 0.07 (0.26) | 0.05 (0.22) | 0.03 (0.16) | <.0001 | <.0001 |
| **Proportion of organic food**  **in the diet** | 0.38 (0.26) | 0.23 (0.20) | 0.20 (0.18) | 0.18 (0.16) | 0.14 (0.14) | <.0001 | <.0001 |

All quantitative variables are presented as mean (SD).
^a^: P-value for Chi-square comparisons or linear regressions as appropriate across quintiles.
NMF: Non-negative Matrix Factorization
sPNNS GS2: simplified Programme National Nutrition Santé Guideline Score 2
PANdiet: Diet Quality Index Based on the Probability of Adequate Nutrient Intake

Supplementary Table 3: Characteristics of quintiles for NMF Component 2, NutriNet-Santé Study, 2014 (N=33,013)

| **NMF Component 2** | **Quintile 1** | **Quintile 2** | **Quintile 3** | **Quintile 4** | **Quintile 5** | **P-value**  **for trend** | **P^a^** |
| --- | --- | --- | --- | --- | --- | --- | --- |
| N | 6602 | 6603 | 6603 | 6603 | 6602 |  |  |
| **Woman**, % | 76.42 | 75.19 | 73.71 | 75.13 | 81.37 | <.0001 | <.0001 |
| **Age**, years, mean (SD) | 49.58  (14.11) | 50.00  (14.01) | 52.40  (13.91) | 54.83  (13.47) | 57.82  (12.67) | <.0001 | <.0001 |
| **Monthly income**  **per household unit**, % |  |  |  |  |  | <.0001 | <.0001 |
| <€1200 | 13.95 | 11.96 | 10.93 | 10.40 | 10.66 |  |  |
| €1200-1800 | 24.34 | 24.81 | 22.32 | 21.72 | 21.77 |  |  |
| €1800-2700 | 26.67 | 26.96 | 27.78 | 27.38 | 27.29 |  |  |
| >€2700 | 29.07 | 31.20 | 33.03 | 34.30 | 32.13 |  |  |
| Unwilling to answer | 5.97 | 5.07 | 5.94 | 6.19 | 8.15 |  |  |
| **Educational level**, % |  |  |  |  |  | <.0001 | <.0001 |
| Less than high-school diploma | 16.57 | 17.36 | 18.49 | 22.19 | 27.23 |  |  |
| High school diploma | 14.13 | 14.46 | 14.13 | 14.45 | 15.84 |  |  |
| Post Graduate | 69.30 | 68.18 | 67.38 | 63.37 | 56.92 |  |  |
| **Occupational status**, % |  |  |  |  |  | <.0001 | <.0001 |
| Employee, manual worker | 16.39 | 15.70 | 14.18 | 13.54 | 13.90 |  |  |
| Intermediate profession | 15.87 | 17.39 | 16.43 | 14.87 | 12.01 |  |  |
| Managerial or intellectual  profession | 24.96 | 26.19 | 22.91 | 19.93 | 14.13 |  |  |
| Retired | 26.45 | 27.50 | 33.95 | 39.24 | 48.27 |  |  |
| Self-employed, farmer | 2.80 | 2.15 | 1.59 | 1.73 | 1.21 |  |  |
| Unemployed or never employed | 13.53 | 11.07 | 10.93 | 10.69 | 10.47 |  |  |
| **Marital status, %** |  |  |  |  |  | <.0001 | <.0001 |
| Single | 15.78 | 13.77 | 12.51 | 11.10 | 10.75 |  |  |
| Divorced or separated | 9.30 | 8.95 | 9.42 | 9.62 | 10.97 |  |  |
| Cohabiting | 21.90 | 22.02 | 18.49 | 16.02 | 12.97 |  |  |
| Married | 50.23 | 52.61 | 56.44 | 60.11 | 60.89 |  |  |
| Widowed | 2.79 | 2.65 | 3.13 | 3.15 | 4.42 |  |  |
| **Place of residence**, % |  |  |  |  |  | <.0001 | <.0001 |
| Rural community | 25.80 | 23.37 | 22.16 | 21.46 | 19.02 |  |  |
| Urban unit with a population  <20,000 inhabitants | 16.15 | 15.63 | 15.14 | 14.95 | 14.86 |  |  |
| Urban unit with a population  between 20,000 and 200,000 | 17.89 | 17.81 | 17.89 | 19.43 | 19.54 |  |  |
| Urban unit with a population  >200,000 inhabitants | 40.17 | 43.19 | 44.81 | 44.16 | 46.58 |  |  |
| **Smoking habits,** % |  |  |  |  |  | 0.21 | <.0001 |
| Current smoker | 11.74 | 12.93 | 11.46 | 10.40 | 8.83 |  |  |
| Former smoker | 36.73 | 38.24 | 40.21 | 41.54 | 41.79 |  |  |
| Never smoker | 51.53 | 48.83 | 48.33 | 48.05 | 49.38 |  |  |
| **Body Mass Index >25 kg/m^2^**, % | 32.23 | 36.09 | 34.64 | 32.36 | 28.13 | <.0001 | <.0001 |
| **Physical activity**, % |  |  |  |  |  | 0.47 | <.0001 |
| High | 33.14 | 29.74 | 30.33 | 34.11 | 39.02 |  |  |
| Moderate | 36.00 | 38.82 | 38.03 | 37.01 | 34.08 |  |  |
| Low | 20.98 | 21.46 | 20.75 | 18.08 | 14.68 |  |  |
| Missing data | 9.88 | 9.98 | 10.89 | 10.80 | 12.22 |  |  |
| **Family history of diabetes**, % | 20.25 | 19.84 | 21.38 | 20.82 | 22.07 | 0.01 | 0.004 |

^a^ : P-value for Chi-square comparisons or linear regressions as appropriate across quintiles.
NMF : Non-negative Matrix Factorization

Supplementary Table 4 : Nutritional characteristics of the participants across quintiles of NMF component 2, NutriNet-Santé Study, 2014 (N=33,013)

| **NMF Component 2** | **Quintile 1** | **Quintile2** | **Quintile 3** | **Quintile 4** | **Quintile 5** | **P-value  for trend** | **P-value^a^** |
| --- | --- | --- | --- | --- | --- | --- | --- |
| **N** | 6602 | 6603 | 6603 | 6603 | 6602 |  |  |
| **Energy intake without**  **alcohol,** kcal/day | 1762 (574) | 1797 (560) | 1906 (582) | 2019 (605) | 2196 (632) | <.0001 | <.0001 |
| **Ethanol**, grams/day | 6.09 (8.89) | 7.87 (10.91) | 8.72 (12.28) | 9.91 (3.99) | 9.19 (14.01) | <.0001 | <.0001 |
| **sPNNS-GS2 score** | 3.40 (3.18) | 2.51 (3.28) | 2.44 (3.42) | 2.52 (3.56) | 3.01 (3.50) | <.0001 | <.0001 |
| **Provegetarian score** | 37.30 (6.37) | 35.48 (5.76) | 35.35 (5.59) | 35.74 (5.60) | 36.42 (5.67) | <.0001 | <.0001 |
|  |  |  |  |  |  |  |  |
| **Special diet,** % |  |  |  |  |  |  |  |
| Omnivorous | 91.15 | 96.06 | 96.56 | 96.82 | 96.26 | <.0001 | <.0001 |
| Pesco-Vegetarian | 2.86 | 1.36 | 1.47 | 1.48 | 1.95 |  |  |
| Vegetarian | 3.89 | 1.83 | 1.42 | 1.08 | 1.15 |  |  |
| Vegan | 2.09 | 0.74 | 0.55 | 0.62 | 0.64 |  |  |
|  |  |  |  |  |  |  |  |
| **PANDiet score** (/100) | 65.30 (8.05) | 64.21 (7.56) | 64.68 (7.51) | 65.41 (7.88) | 65.51 (8.39) | <.0001 | <.0001 |
| **Carbohydrates**  (% of alcohol free  energy intake) | 40.15 (7.94) | 39.07 (7.30) | 38.98 (7.24) | 39.28 (7.24) | 41.11 (7.63) | <.0001 | <.0001 |
| **Lipids** (% of alcohol-  free energy intake) | 41.59 (7.55) | 41.73 (6.73) | 41.54 (6.85) | 41.25 (6.96) | 39.77 (7.29) | <.0001 | <.0001 |
| **Protein** (% of alcohol-free energy intake) | 17.86 (3.86) | 18.84 (3.61) | 19.11 (3.61) | 19.09 (3.52) | 18.67 (3.68) | <.0001 | <.0001 |
| Plant /  total protein ratio | 0.39 (0.19) | 0.33 (0.14) | 0.32 (0.13) | 0.32 (0.13) | 0.34 (0.13) | <.0001 | <.0001 |
| **Proportion of individuals with organic food in the diet ≥ 50%** | 0.36 (0.48) | 0.12 (0.33) | 0.07 (0.25) | 0.04 (0.21) | 0.03 (0.17) | <.0001 | <.0001 |
| **Proportion of organic food**  **in the diet** | 0.39 (0.25) | 0.24 (0.20) | 0.19 (0.18) | 0.16 (0.16) | 0.14 (0.15) | <.0001 | <.0001 |

^a^: P-value for Chi-square comparisons or linear regressions as appropriate across quintiles.
NMF : Non-negative Matrix Factorization
sPNNS GS2: simplified Programme National Nutrition Santé Guideline Score 2
PANdiet: Diet Quality Index Based on the Probability of Adequate Nutrient Intake

Supplementary Table 5: Characteristics of quintiles for NMF Component 3, NutriNet-Santé Study, 2014 (N=33,013)

| **NMF Component 3** | **Quintile 1** | **Quintile 2** | **Quintile 3** | **Quintile 4** | **Quintile 5** | **P-value**  **for trend** | **P^a^** |
| --- | --- | --- | --- | --- | --- | --- | --- |
| N | 6602 | 6603 | 6603 | 6603 | 6602 |  |  |
| **Woman**, % | 65,15 | 72,66 | 78,48 | 81,81 | 83,72 | <.0001 | <.0001 |
| **Age**, years, mean (SD) | 51.94  (15.03) | 51.00  (14.66) | 52.26  (14.09) | 53.67  (13.24) | 55.76  (12.24) | <.0001 | <.0001 |
| **Monthly income**  **per household unit**, % |  |  |  |  |  | 0.01 | <.0001 |
| <€1200 | 13.51 | 12.37 | 10.84 | 10.40 | 10.78 |  |  |
| €1200-1800 | 24.46 | 23.14 | 22.75 | 22.22 | 22.39 |  |  |
| €1800-2700 | 26.20 | 27.20 | 27.87 | 27.28 | 27.54 |  |  |
| >€2700 | 29.79 | 31.00 | 32.39 | 34.26 | 32.28 |  |  |
| Unwilling to answer | 6.03 | 6.29 | 6.15 | 5.85 | 7.01 |  |  |
| **Educational level**, % |  |  |  |  |  | <.0001 | <.0001 |
| Less than high-school diploma | 23.83 | 19.29 | 19.05 | 18.45 | 21.22 |  |  |
| High school diploma | 16.33 | 14.69 | 13.08 | 14.80 | 14.12 |  |  |
| Post Graduate | 59.85 | 66.02 | 67.86 | 66.76 | 64.66 |  |  |
| **Occupational status**, % |  |  |  |  |  | <.0001 | <.0001 |
| Employee, manual worker | 17.56 | 16.45 | 14.07 | 13.13 | 12.51 |  |  |
| Intermediate profession | 14.13 | 16.31 | 16.52 | 15.75 | 13.86 |  |  |
| Managerial or intellectual  profession | 19.62 | 22.81 | 23.73 | 22.88 | 19.09 |  |  |
| Retired | 34.81 | 31.32 | 32.94 | 35.82 | 40.53 |  |  |
| Self-employed, farmer | 1.58 | 1.86 | 1.88 | 1.68 | 2.48 |  |  |
| Unemployed or never employed | 12.31 | 11.25 | 10.86 | 10.74 | 11.53 |  |  |
| **Marital status, %** |  |  |  |  |  | <.0001 | <.0001 |
| Single | 15.21 | 15.33 | 12.68 | 11.10 | 9.60 |  |  |
| Divorced or separated | 9.41 | 9.42 | 9.56 | 10.01 | 9.86 |  |  |
| Cohabiting | 18.81 | 19.84 | 19.35 | 17.61 | 15.78 |  |  |
| Married | 53.44 | 52.32 | 55.02 | 57.81 | 61.69 |  |  |
| Widowed | 3.14 | 3.09 | 3.39 | 3.47 | 3.06 |  |  |
| **Place of residence**, % |  |  |  |  |  | <.0001 | <.0001 |
| Rural community | 20.19 | 20.58 | 20.91 | 22.28 | 27.84 |  |  |
| Urban unit with a population  <20,000 inhabitants | 15.13 | 13.68 | 14.69 | 15.99 | 17.24 |  |  |
| Urban unit with a population  between 20,000 and 200,000 | 18.24 | 18.37 | 18.54 | 18.07 | 19.34 |  |  |
| Urban unit with a population  >200,000 inhabitants | 46.44 | 47.37 | 45.86 | 43.66 | 35.58 |  |  |
| **Smoking habits,** % |  |  |  |  |  | 0.0001 | <.0001 |
| Current smoker | 12.83 | 12.31 | 11.06 | 10.16 | 9.01 |  |  |
| Former smoker | 38.20 | 38.50 | 39.38 | 40.59 | 41.85 |  |  |
| Never smoker | 48.97 | 49.19 | 49.57 | 49.25 | 49.14 |  |  |
| **Body Mass Index >25 kg/m^2^**, % | 41.06 | 38.68 | 32.92 | 28.53 | 22.25 | <.0001 | <.0001 |
| **Physical activity**, % |  |  |  |  |  |  |  |
| High | 29.48 | 29.73 | 31.18 | 34.51 | 41.44 | <.0001 | <.0001 |
| Moderate | 34.53 | 37.86 | 38.89 | 37.54 | 35.11 |  |  |
| Low | 24.55 | 20.93 | 19.28 | 17.76 | 13.42 |  |  |
| Missing data | 11.44 | 11.48 | 10.65 | 10.18 | 10.03 |  |  |
| **Family history of diabetes**, % | 21.31 | 21.10 | 20.26 | 20.43 | 21.27 | 0.43 | 0.63 |

^a:^ P-value for Chi-square comparisons or linear regressions as appropriate across quintiles.
NMF: Non-negative Matrix Factorization

Supplementary Table 6 : Nutritional characteristics of the participants across quintiles of NMF component 3, NutriNet-Santé Study, 2014 (N=33,013)

| **NMF Component 3** | **Quintile 1** | **Quintile2** | **Quintile 3** | **Quintile 4** | **Quintile 5** | **P-value  for trend** | **P-value^a^** |
| --- | --- | --- | --- | --- | --- | --- | --- |
| **N** | 6602 | 6603 | 6603 | 6603 | 6602 |  |  |
| **Energy intake without**  **Alcohol,** kcal/day | 1911  (640) | 1889  (612) | 1895  (588) | 1935  (594) | 2052  (611) | <.0001 | <.0001 |
| **Ethanol**, grams/day | 9.53 (5.72) | 8.07 (11.83) | 7.97 (11.00) | 8.19 (11.23) | 8.04 (10.66) | <.0001 | <.0001 |
| **sPNNS-GS2 score** | 1.74 (3.55) | 2.51 (3.34) | 2.87 (3.28) | 3.13 (3.30) | 3.62 (3.29) | <.0001 | <.0001 |
| **Provegetarian score** | 34.21 (5.38) | 35.08 (5.53) | 35.81 (5.51) | 36.59 (5.70) | 38.62 (6.12) | <.0001 | <.0001 |
|  |  |  |  |  |  |  |  |
| **Special diet,** % |  |  |  |  |  | <.0001 | <.0001 |
| Omnivorous | 97.26 | 96.24 | 96.08 | 95.27 | 92.00 |  |  |
| Pesco-Vegetarian | 0.77 | 1.45 | 1.61 | 1.91 | 3.39 |  |  |
| Vegetarian | 1.35 | 1.59 | 1.70 | 1.85 | 2.89 |  |  |
| Vegan | 0.62 | 0.71 | 0.62 | 0.97 | 1.71 |  |  |
|  |  |  |  |  |  |  |  |
| **PANDiet score** (/100) | 63.31 (7.91) | 64.30 (7.69) | 64.99 (7.67) | 65.56 (7.68) | 66.95 (8.05) | <.0001 | <.0001 |
| **Carbohydrates**  (% of alcohol free  energy intake) | 39.79 (7.82) | 39.96 (7.38) | 39.68 (7.39) | 39.60 (7.26) | 39.57 (7.72) | <.0001 | 0.02 |
| **Lipids** (% of alcohol-  free energy intake) | 40.43 (7.11) | 40.60 (6.90) | 41.15 (6.97) | 41.51 (6.91) | 42.19 (7.54) | <.0001 | <.0001 |
| **Protein** (% of alcohol-free energy intake) | 19.42 (3.81) | 19.05 (3.67) | 18.77 (3.52) | 18.49 (3.58) | 17.84 (3.65) | <.0001 | <.0001 |
| Plant /  total protein ratio | 0.30 (0.12) | 0.32 (0.14) | 0.33 (0.13) | 0.35 (0.14) | 0.40 (0.17) | <.0001 | <.0001 |
| **Proportion of individuals with organic food**  **in the diet ≥ 50%** | 0.02 (0.14) | 0.06 (0.24) | 0.09 (0.29) | 0.14 (0.35) | 0.31 (0.46) | <.0001 | <.0001 |
| **Proportion of organic**  **food** **in the diet** | 0.08 (0.13) | 0.17 (0.17) | 0.21 (0.19) | 0.27 (0.20) | 0.38 (0.22) | <.0001 | <.0001 |

^a^: P-value for Chi-square comparisons or linear regressions as appropriate across quintiles.
NMF: Non-negative Matrix Factorization
sPNNS GS2: simplified Programme National Nutrition Santé Guideline Score 2
PANdiet: Diet Quality Index Based on the Probability of Adequate Nutrient Intake

Supplementary Table 7: Characteristics of quintiles for NMF Component 4, NutriNet-Santé Study, 2014 (N=33,013)

| **NMF Component 4** | **Quintile 1** | **Quintile 2** | **Quintile 3** | **Quintile 4** | **Quintile 5** | **P-value**  **for trend** | **P^a^** |
| --- | --- | --- | --- | --- | --- | --- | --- |
| N | 6602 | 6603 | 6603 | 6603 | 6602 |  |  |
| **Woman**, % | 62.78 | 71.60 | 78.15 | 80.87 | 88.41 | <.0001 | <.0001 |
| **Age**, years, mean (SD) | 55.86  (13.18) | 52.23  (14.14) | 51.29  (14.46) | 52.58  (14.15) | 52.67  (13.55) | <.0001 | <.0001 |
| **Monthly income**  **per household unit**, % |  |  |  |  |  | <.0001 | <.0001 |
| <€1200 | 12.60 | 11.80 | 12.16 | 10.93 | 10.42 |  |  |
| €1200-1800 | 24.51 | 24.78 | 22.19 | 22.31 | 21.18 |  |  |
| €1800-2700 | 27.13 | 26.88 | 27.38 | 27.67 | 27.02 |  |  |
| >€2700 | 29.20 | 30.88 | 32.15 | 32.92 | 34.57 |  |  |
| Unwilling to answer | 6.56 | 5.66 | 6.12 | 6.16 | 6.82 |  |  |
| **Educational level**, % |  |  |  |  |  | <.0001 | <.0001 |
| Less than high-school diploma | 29.22 | 20.35 | 17.61 | 18.73 | 15.92 |  |  |
| High school diploma | 15.90 | 14.86 | 14.52 | 14.28 | 13.45 |  |  |
| Post Graduate | 54.88 | 64.79 | 67.86 | 66.98 | 70.63 |  |  |
| **Occupational status**, % |  |  |  |  |  | <.0001 | <.0001 |
| Employee, manual worker | 14.86 | 15.40 | 15.02 | 14.58 | 13.84 |  |  |
| Intermediate profession | 11.74 | 15.83 | 15.60 | 16.83 | 16.59 |  |  |
| Managerial or intellectual  profession | 16.24 | 21.75 | 23.76 | 22.20 | 24.17 |  |  |
| Retired | 44.20 | 33.67 | 31.46 | 34.09 | 32.01 |  |  |
| Self-employed, farmer | 2.15 | 1.56 | 2.01 | 1.86 | 1.89 |  |  |
| Unemployed or never employed | 10.81 | 11.80 | 12.15 | 10.43 | 11.50 |  |  |
| **Marital status, %** |  |  |  |  |  | <.0001 | <.0001 |
| Single | 10.78 | 13.48 | 14.30 | 12.63 | 12.72 |  |  |
| Divorced or separated | 9.50 | 9.33 | 8.98 | 9.62 | 10.83 |  |  |
| Cohabiting | 14.69 | 19.17 | 21.14 | 18.90 | 17.49 |  |  |
| Married | 61.71 | 54.81 | 52.43 | 55.70 | 55.63 |  |  |
| Widowed | 3.32 | 3.21 | 3.15 | 3.15 | 3.32 |  |  |
| **Place of residence**, % |  |  |  |  |  | <.0001 | <.0001 |
| Rural community | 25.42 | 23.22 | 21.90 | 21.11 | 20.16 |  |  |
| Urban unit with a population  <20,000 inhabitants | 17.21 | 15.45 | 14.58 | 15.13 | 14.36 |  |  |
| Urban unit with a population  between 20,000 and 200,000 | 18.49 | 18.46 | 18.87 | 18.23 | 18.49 |  |  |
| Urban unit with a population  >200,000 inhabitants | 38.88 | 42.87 | 44.65 | 45.52 | 46.99 |  |  |
| **Smoking habits,** % |  |  |  |  |  | <.0001 | <.0001 |
| Current smoker | 12.12 | 12.19 | 11.34 | 9.72 | 10.00 |  |  |
| Former smoker | 42.23 | 38.79 | 38.15 | 39.83 | 39.52 |  |  |
| Never smoker | 45.65 | 49.02 | 50.51 | 50.45 | 50.48 |  |  |
| **Body Mass Index >25 kg/m^2^**, % | 42.67 | 36.07 | 29.46 | 32.71 | 22.54 | <.0001 | <.0001 |
| **Physical activity**, % |  |  |  |  |  | 0.24 | <.0001 |
| High | 35.28 | 32.79 | 33.00 | 32.18 | 33.10 |  |  |
| Moderate | 33.13 | 35.98 | 38.01 | 38.33 | 38.49 |  |  |
| Low | 20.07 | 20.69 | 18.69 | 19.04 | 17.46 |  |  |
| Missing data | 11.53 | 10.54 | 10.30 | 10.45 | 10.95 |  |  |
| **Family history of diabetes**, % | 22.13 | 20.76 | 20.55 | 20.78 | 20.15 | 0.06 | 0.01 |

^a^P-value for Chi-square comparisons or linear regressions as appropriate across quintiles.
NMF: Non-negative Matrix Factorization

Supplementary Table 8 : Nutritional characteristics of the participants across quintiles of NMF component 4, NutriNet-Santé Study, 2014 (N=33,013)

| **NMF Component 4** | **Quintile 1** | **Quintile2** | **Quintile 3** | **Quintile 4** | **Quintile 5** | **P-value  for trend** | **P-value^a^** |
| --- | --- | --- | --- | --- | --- | --- | --- |
| **N** | 6602 | 6603 | 6603 | 6603 | 6602 |  |  |
| **Energy intake without**  **Alcohol,** kcal/day | 1922 (629) | 1938 (617) | 1973 (621) | 1935 (607) | 1913 (584) | <.0001 | <.0001 |
| **Ethanol**, grams/day | 10.60 (16.27) | 8.58 (12.33) | 7.57 (10.60) | 7.77 (10.57) | 7.27 (10.06) | <.0001 | <.0001 |
| **sPNNS-GS2 score** | 2.27 (3.60) | 2.62 (3.50) | 2.97 (3.34) | 2.91 (3.32) | 3.11 (3.23) | <.0001 | <.0001 |
| **Provegetarian score** | 35.37 (5.93) | 36.13 (5.96) | 36.66 (6.12) | 36.06 (5.64) | 36.08 (5.51) | <.0001 | <.0001 |
|  |  |  |  |  |  |  |  |
| **Special diet,** % |  |  |  |  |  | 0.39 | <.0001 |
| Omnivorous | 96.18 | 95.23 | 93.65 | 96.03 | 95.76 |  |  |
| Pesco-Vegetarian | 1.38 | 1.68 | 2.35 | 1.65 | 2.08 |  |  |
| Vegetarian | 1.54 | 1.97 | 2.70 | 1.54 | 1.62 |  |  |
| Vegan | 0.89 | 1.12 | 1.30 | 0.77 | 0.55 |  |  |
|  |  |  |  |  |  |  |  |
| **PANDiet score** (/100) | 64.43 (8.06) | 64.93 (7.83) | 65.47 (7.86) | 64.97 (7.91) | 65.30 (7.78) | <.0001 | <.0001 |
| **Carbohydrates**  (% of alcohol free  energy intake) | 39.20 (7.81) | 39.55 (7.34) | 40.22 (7.50) | 39.74 (7.40) | 39.90 (7.49) | <.0001 | <.0001 |
| **Lipids** (% of alcohol-  free energy intake) | 41.25 (7.31) | 41.44 (7.04) | 41.03 (7.08) | 41.16 (7.05) | 41.01 (7.11) | <.0001 | 0.003 |
| **Protein** (% of alcohol-free energy intake) | 19.22 (3.79) | 18.64 (3.79) | 18.34 (3.68) | 18.70 (3.54) | 18.67 (3.57) | <.0001 | <.0001 |
| Plant /  total protein ratio | 0.32 (0.15) | 0.34 (0.16) | 0.36 (0.16) | 0.33 (0.14) | 0.34 (0.13) | <.0001 | <.0001 |
| **Proportion of individuals with organic food in the diet ≥ 50%** | 0.13 (0.34) | 0.17 (0.37) | 0.19 (0.39) | 0.09 (0.29) | 0.04 (0.21) | <.0001 | <.0001 |
| **Proportion of organic food**  **in the diet** | 0.21 (0.22) | 0.24 (0.23) | 0.27 (0.23) | 0.22 (0.19) | 0.17 (0.16) | <.0001 | <.0001 |

^a^: p-value for Chi-square comparisons across quintiles
NMF: Non-negative Matrix Factorization
sPNNS GS2: simplified Programme National Nutrition Santé Guideline Score 2
PANdiet: Diet Quality Index Based on the Probability of Adequate Nutrient Intake

Supplementary Table 9: Estimated dietary pesticide exposure across NMF Components 1 and 2 quintiles (in μg/kg of weight/day), NutriNet-Santé Study, 2014 (N=33,013)

| **NMF Component 1** | **Quintile 1  N=6602** | | **Quintile 2**  **N=6603** | | **Quintile 3  N=6603** | | **Quintile 4  N=6603** | | **Quintile 5**  **N=6602** | |
| --- | --- | --- | --- | --- | --- | --- | --- | --- | --- | --- |
| **Variable** | **Mean** | **SD** | **Mean** | **SD** | **Mean** | **SD** | **Mean** | **SD** | **Mean** | **SD** |
| **Acetamiprid** | 0.0317 | 0.0587 | 0.0444 | 0.0607 | 0.0480 | 0.0622 | 0.0562 | 0.0719 | 0.0724 | 0.0820 |
| **Anthraquinone** | 0.0004 | 0.0012 | 0.0006 | 0.0013 | 0.0006 | 0.0017 | 0.0006 | 0.0016 | 0.0007 | 0.0020 |
| **Azadirachtin** | 0.0005 | 0.0006 | 0.0003 | 0.0004 | 0.0003 | 0.0003 | 0.0003 | 0.0004 | 0.0003 | 0.0004 |
| **Azoxystrobin** | 0.0150 | 0.0241 | 0.0285 | 0.0199 | 0.0403 | 0.0293 | 0.0515 | 0.0401 | 0.0787 | 0.0765 |
| **Boscalid** | 0.0604 | 0.0941 | 0.0885 | 0.0752 | 0.1089 | 0.0836 | 0.1291 | 0.0941 | 0.1823 | 0.1312 |
| **Carbendazim** | 0.0339 | 0.0467 | 0.0431 | 0.0464 | 0.0464 | 0.0468 | 0.0534 | 0.0533 | 0.0677 | 0.0617 |
| **Chlorpropham** | 0.0369 | 0.0522 | 0.0564 | 0.0592 | 0.0641 | 0.0619 | 0.0703 | 0.0678 | 0.0815 | 0.0757 |
| **Chlorpyrifos** | 0.0269 | 0.0343 | 0.0420 | 0.0390 | 0.0544 | 0.0389 | 0.0736 | 0.0426 | 0.1311 | 0.0738 |
| **Cypermethrin** | 0.0520 | 0.0849 | 0.0658 | 0.0879 | 0.0699 | 0.0891 | 0.0823 | 0.1026 | 0.1054 | 0.1168 |
| **Cyprodinil** | 0.0363 | 0.0563 | 0.0520 | 0.0495 | 0.0654 | 0.0746 | 0.0771 | 0.0652 | 0.1140 | 0.1037 |
| **Difenoconazole** | 0.0086 | 0.0142 | 0.0131 | 0.0113 | 0.0157 | 0.0145 | 0.0190 | 0.0153 | 0.0264 | 0.0198 |
| **Dimethoate Ometoate** | 0.0054 | 0.0091 | 0.0073 | 0.0092 | 0.0083 | 0.0105 | 0.0097 | 0.0127 | 0.0134 | 0.0145 |
| **Fenhexamid** | 0.0415 | 0.0734 | 0.0660 | 0.0888 | 0.0852 | 0.1308 | 0.1049 | 0.1271 | 0.1525 | 0.1755 |
| **Glyphosate** | 0.0021 | 0.0042 | 0.0031 | 0.0042 | 0.0036 | 0.0044 | 0.0040 | 0.0048 | 0.0049 | 0.0060 |
| **Imazalil** | 0.0635 | 0.0447 | 0.2429 | 0.0646 | 0.4831 | 0.0971 | 0.8746 | 0.1679 | 2.0941 | 1.2853 |
| **Imidacloprid** | 0.0370 | 0.0431 | 0.0526 | 0.0466 | 0.0684 | 0.0541 | 0.1009 | 0.0680 | 0.1384 | 0.0982 |
| **Iprodione** | 0.0596 | 0.1179 | 0.0931 | 0.1146 | 0.1196 | 0.1239 | 0.1470 | 0.1393 | 0.2256 | 0.2112 |
| **lambda Cyhalothrin** | 0.0047 | 0.0069 | 0.0073 | 0.0078 | 0.0091 | 0.0075 | 0.0109 | 0.0084 | 0.0165 | 0.0126 |
| **Malathion** | 0.0001 | 0.0002 | 0.0002 | 0.0004 | 0.0003 | 0.0003 | 0.0003 | 0.0003 | 0.0006 | 0.0006 |
| **Methamidophos** | 0.0002 | 0.0003 | 0.0003 | 0.0003 | 0.0003 | 0.0004 | 0.0003 | 0.0004 | 0.0003 | 0.0004 |
| **Profenofos** | 0.0000 | 0.0000 | 0.0000 | 0.0000 | 0.0000 | 0.0000 | 0.0001 | 0.0000 | 0.0001 | 0.0001 |
| **Pyrethrins** | 0.0021 | 0.0020 | 0.0020 | 0.0018 | 0.0021 | 0.0018 | 0.0022 | 0.0018 | 0.0023 | 0.0022 |
| **Spinosad** | 0.1882 | 0.2316 | 0.1300 | 0.1521 | 0.1278 | 0.1499 | 0.1305 | 0.1467 | 0.1321 | 0.1542 |
| **Tebuconazole** | 0.0144 | 0.0249 | 0.0224 | 0.0276 | 0.0292 | 0.0328 | 0.0360 | 0.0382 | 0.0560 | 0.0547 |
| **Thiabendazole** | 0.0305 | 0.0217 | 0.0976 | 0.0300 | 0.1835 | 0.0463 | 0.3321 | 0.0822 | 0.7087 | 0.3964 |
|  |  |  |  |  |  |  |  |  |  |  |
| **NMF Component 2** | **Quintile 1  N=6602** | | **Quintile 2  N=6603** | | **Quintile 3  N=6603** | | **Quintile 4  N=6603** | | **Quintile 5  N=6602** | |
| **Variable** | **Mean** | **SD** | **Mean** | **SD** | **Mean** | **SD** | **Mean** | **SD** | **Mean** | **SD** |
| **Acetamiprid** | 0.0297 | 0.0538 | 0.0403 | 0.0591 | 0.0484 | 0.0661 | 0.0558 | 0.0695 | 0.0785 | 0.0827 |
| **Anthraquinone** | 0.0004 | 0.0011 | 0.0006 | 0.0012 | 0.0006 | 0.0015 | 0.0007 | 0.0022 | 0.0006 | 0.0016 |
| **Azadirachtin** | 0.0004 | 0.0006 | 0.0003 | 0.0003 | 0.0003 | 0.0004 | 0.0003 | 0.0004 | 0.0004 | 0.0005 |
| **Azoxystrobin** | 0.0167 | 0.0418 | 0.0270 | 0.0261 | 0.0378 | 0.0302 | 0.0515 | 0.0398 | 0.0809 | 0.0652 |
| **Boscalid** | 0.0317 | 0.0283 | 0.0606 | 0.0237 | 0.0916 | 0.0292 | 0.1336 | 0.0407 | 0.2517 | 0.1506 |
| **Carbendazim** | 0.0318 | 0.0402 | 0.0396 | 0.0443 | 0.0470 | 0.0496 | 0.0539 | 0.0528 | 0.0722 | 0.0635 |
| **Chlorpropham** | 0.0218 | 0.0257 | 0.0442 | 0.0338 | 0.0625 | 0.0462 | 0.0793 | 0.0607 | 0.1015 | 0.1005 |
| **Chlorpyrifos** | 0.0428 | 0.0577 | 0.0498 | 0.0434 | 0.0601 | 0.0483 | 0.0718 | 0.0540 | 0.1035 | 0.0726 |
| **Cypermethrin** | 0.0529 | 0.0789 | 0.0631 | 0.0863 | 0.0726 | 0.0966 | 0.0808 | 0.1014 | 0.1060 | 0.1173 |
| **Cyprodinil** | 0.0186 | 0.0144 | 0.0341 | 0.0129 | 0.0514 | 0.0173 | 0.0764 | 0.0250 | 0.1643 | 0.1232 |
| **Difenoconazole** | 0.0066 | 0.0070 | 0.0107 | 0.0075 | 0.0147 | 0.0093 | 0.0195 | 0.0121 | 0.0313 | 0.0254 |
| **Dimethoate Ometoate** | 0.0049 | 0.0078 | 0.0064 | 0.0084 | 0.0079 | 0.0095 | 0.0093 | 0.0101 | 0.0156 | 0.0171 |
| **Fenhexamid** | 0.0193 | 0.0204 | 0.0390 | 0.0251 | 0.0608 | 0.0408 | 0.0981 | 0.0718 | 0.2329 | 0.2183 |
| **Glyphosate** | 0.0017 | 0.0031 | 0.0028 | 0.0042 | 0.0035 | 0.0042 | 0.0044 | 0.0051 | 0.0054 | 0.0064 |
| **Imazalil** | 0.5674 | 1.1893 | 0.5445 | 0.6024 | 0.6469 | 0.6618 | 0.8006 | 0.7458 | 1.1988 | 1.1208 |
| **Imidacloprid** | 0.0758 | 0.0827 | 0.0709 | 0.0680 | 0.0747 | 0.0712 | 0.0776 | 0.0675 | 0.0982 | 0.0791 |
| **Iprodione** | 0.0250 | 0.0268 | 0.0557 | 0.0258 | 0.0899 | 0.0359 | 0.1418 | 0.0528 | 0.3325 | 0.2398 |
| **Lambda Cyhalothrin** | 0.0030 | 0.0030 | 0.0053 | 0.0028 | 0.0076 | 0.0036 | 0.0109 | 0.0046 | 0.0217 | 0.0144 |
| **Malathion** | 0.0002 | 0.0005 | 0.0002 | 0.0002 | 0.0003 | 0.0003 | 0.0003 | 0.0004 | 0.0005 | 0.0005 |
| **Methamidophos** | 0.0002 | 0.0003 | 0.0002 | 0.0003 | 0.0003 | 0.0003 | 0.0003 | 0.0004 | 0.0003 | 0.0004 |
| **Profenofos** | 0.0000 | 0.0001 | 0.0000 | 0.0000 | 0.0000 | 0.0000 | 0.0000 | 0.0000 | 0.0001 | 0.0001 |
| **Pyrethrins** | 0.0021 | 0.0019 | 0.0020 | 0.0017 | 0.0021 | 0.0018 | 0.0022 | 0.0020 | 0.0023 | 0.0022 |
| **Spinosad** | 0.1845 | 0.2244 | 0.1298 | 0.1521 | 0.1267 | 0.1488 | 0.1256 | 0.1480 | 0.1421 | 0.1651 |
| **Tebuconazole** | 0.0084 | 0.0076 | 0.0153 | 0.0074 | 0.0225 | 0.0104 | 0.0331 | 0.0153 | 0.0786 | 0.0661 |
| **Thiabendazole** | 0.2082 | 0.3823 | 0.2057 | 0.2083 | 0.2401 | 0.2273 | 0.2882 | 0.2454 | 0.4102 | 0.3578 |

NMF: Non-Negative Matrix Factorization; SD: Standard Deviation

Supplementary Table 10: Estimated dietary pesticide exposure across NMF Components 3 and 4 quintiles (in μg/kg of weight/day), NutriNet-Santé Study, 2014 (N=33,013)

| **NMF Component 3** | **Quintile 1  N=6602** | | **Quintile 2  N=6603** | | **Quintile 3  N=6603** | | **Quintile 4 N=6603** | | **Quintile 5  N=6602** | |
| --- | --- | --- | --- | --- | --- | --- | --- | --- | --- | --- |
| **Variable** | **Mean** | **SD** | **Mean** | **SD** | **Mean** | **SD** | **Mean** | **SD** | **Mean** | **SD** |
| **Acetamiprid** | 0.0150 | 0.0147 | 0.0290 | 0.0218 | 0.0505 | 0.0375 | 0.0720 | 0.0645 | 0.0863 | 0.1185 |
| **Anthraquinone** | 0.0006 | 0.0017 | 0.0006 | 0.0017 | 0.0006 | 0.0012 | 0.0006 | 0.0014 | 0.0006 | 0.0018 |
| **Azadirachtin** | 0.0001 | 0.0001 | 0.0002 | 0.0002 | 0.0003 | 0.0003 | 0.0004 | 0.0004 | 0.0007 | 0.0007 |
| **Azoxystrobin** | 0.0495 | 0.0524 | 0.0438 | 0.0449 | 0.0429 | 0.0444 | 0.0411 | 0.0425 | 0.0367 | 0.0549 |
| **Boscalid** | 0.1191 | 0.1010 | 0.1133 | 0.1075 | 0.1142 | 0.0991 | 0.1147 | 0.0959 | 0.1078 | 0.1233 |
| **Carbendazim** | 0.0212 | 0.0152 | 0.0324 | 0.0203 | 0.0488 | 0.0302 | 0.0653 | 0.0492 | 0.0768 | 0.0880 |
| **Chlorpropham** | 0.0853 | 0.0897 | 0.0665 | 0.0609 | 0.0619 | 0.0571 | 0.0554 | 0.0551 | 0.0401 | 0.0487 |
| **Chlorpyrifos** | 0.0485 | 0.0468 | 0.0568 | 0.0493 | 0.0676 | 0.0519 | 0.0760 | 0.0604 | 0.0792 | 0.0798 |
| **Cypermethrin** | 0.0197 | 0.0139 | 0.0421 | 0.0284 | 0.0746 | 0.0519 | 0.1073 | 0.0911 | 0.1317 | 0.1681 |
| **Cyprodinil** | 0.0706 | 0.0884 | 0.0704 | 0.0792 | 0.0694 | 0.0711 | 0.0692 | 0.0678 | 0.0653 | 0.0769 |
| **Difenoconazole** | 0.0148 | 0.0162 | 0.0152 | 0.0154 | 0.0165 | 0.0138 | 0.0179 | 0.0143 | 0.0183 | 0.0210 |
| **Dimethoate Ometoate** | 0.0037 | 0.0083 | 0.0057 | 0.0066 | 0.0088 | 0.0079 | 0.0118 | 0.0107 | 0.0141 | 0.0179 |
| **Fenhexamid** | 0.0981 | 0.1606 | 0.0891 | 0.1200 | 0.0912 | 0.1256 | 0.0888 | 0.1183 | 0.0829 | 0.1199 |
| **Glyphosate** | 0.0040 | 0.0057 | 0.0037 | 0.0053 | 0.0036 | 0.0046 | 0.0035 | 0.0046 | 0.0031 | 0.0042 |
| **Imazalil** | 0.7828 | 0.9235 | 0.7838 | 0.9615 | 0.7822 | 0.9213 | 0.7341 | 0.8817 | 0.6753 | 0.9514 |
| **Imidacloprid** | 0.0621 | 0.0698 | 0.0699 | 0.0657 | 0.0806 | 0.0683 | 0.0889 | 0.0728 | 0.0959 | 0.0890 |
| **Iprodione** | 0.1198 | 0.1172 | 0.1332 | 0.1564 | 0.1338 | 0.1606 | 0.1320 | 0.1573 | 0.1262 | 0.1827 |
| **lambda Cyhalothrin** | 0.0089 | 0.0073 | 0.0098 | 0.0094 | 0.0100 | 0.0097 | 0.0101 | 0.0102 | 0.0097 | 0.0115 |
| **Malathion** | 0.0003 | 0.0004 | 0.0003 | 0.0003 | 0.0003 | 0.0003 | 0.0003 | 0.0003 | 0.0003 | 0.0006 |
| **Methamidophos** | 0.0003 | 0.0004 | 0.0003 | 0.0004 | 0.0003 | 0.0003 | 0.0003 | 0.0003 | 0.0002 | 0.0003 |
| **Profenofos** | 0.0000 | 0.0000 | 0.0000 | 0.0000 | 0.0000 | 0.0000 | 0.0000 | 0.0000 | 0.0000 | 0.0000 |
| **Pyrethrins** | 0.0020 | 0.0022 | 0.0019 | 0.0017 | 0.0020 | 0.0016 | 0.0021 | 0.0018 | 0.0027 | 0.0021 |
| **Spinosad** | 0.0121 | 0.0086 | 0.0407 | 0.0148 | 0.0879 | 0.0192 | 0.1643 | 0.0330 | 0.4036 | 0.2157 |
| **Tebuconazole** | 0.0291 | 0.0348 | 0.0332 | 0.0391 | 0.0330 | 0.0413 | 0.0320 | 0.0397 | 0.0305 | 0.0433 |
| **Thiabendazole** | 0.2922 | 0.3054 | 0.2816 | 0.3009 | 0.2798 | 0.2915 | 0.2623 | 0.2953 | 0.2365 | 0.3168 |
|  |  |  |  |  |  |  |  |  |  |  |
| **NMF Component 4** | **Quintile 1  N=6602** | | **Quintile 2  N=6603** | | **Quintile 3  N=6603** | | **Quintile 4 N=6603** | | **Quintile 5  N=6602** | |
| **Variable** | **Mean** | **SD** | **Mean** | **SD** | **Mean** | **SD** | **Mean** | **SD** | **Mean** | **SD** |
| **Acetamiprid** | 0.0106 | 0.0112 | 0.0128 | 0.0086 | 0.0227 | 0.0143 | 0.0544 | 0.0208 | 0.1521 | 0.0940 |
| **Anthraquinone** | 0.0003 | 0.0006 | 0.0005 | 0.0009 | 0.0007 | 0.0014 | 0.0007 | 0.0016 | 0.0008 | 0.0025 |
| **Azadirachtin** | 0.0003 | 0.0005 | 0.0003 | 0.0005 | 0.0004 | 0.0004 | 0.0003 | 0.0004 | 0.0003 | 0.0004 |
| **Azoxystrobin** | 0.0438 | 0.0657 | 0.0354 | 0.0364 | 0.0395 | 0.0406 | 0.0446 | 0.0416 | 0.0507 | 0.0499 |
| **Boscalid** | 0.1031 | 0.1116 | 0.0929 | 0.0718 | 0.1100 | 0.0951 | 0.1200 | 0.1036 | 0.1431 | 0.1310 |
| **Carbendazim** | 0.0145 | 0.0113 | 0.0195 | 0.0096 | 0.0312 | 0.0139 | 0.0535 | 0.0167 | 0.1258 | 0.0694 |
| **Chlorpropham** | 0.0661 | 0.0874 | 0.0566 | 0.0563 | 0.0572 | 0.0609 | 0.0620 | 0.0584 | 0.0673 | 0.0590 |
| **Chlorpyrifos** | 0.0385 | 0.0514 | 0.0385 | 0.0312 | 0.0522 | 0.0403 | 0.0725 | 0.0460 | 0.1263 | 0.0726 |
| **Cypermethrin** | 0.0135 | 0.0117 | 0.0202 | 0.0098 | 0.0374 | 0.0164 | 0.0820 | 0.0248 | 0.2223 | 0.1326 |
| **Cyprodinil** | 0.0671 | 0.1048 | 0.0550 | 0.0469 | 0.0660 | 0.0660 | 0.0713 | 0.0686 | 0.0854 | 0.0833 |
| **Difenoconazole** | 0.0114 | 0.0152 | 0.0117 | 0.0107 | 0.0148 | 0.0146 | 0.0176 | 0.0154 | 0.0272 | 0.0195 |
| **Dimethoate Ometoate** | 0.0029 | 0.0049 | 0.0031 | 0.0036 | 0.0051 | 0.0062 | 0.0095 | 0.0072 | 0.0234 | 0.0162 |
| **Fenhexamid** | 0.1027 | 0.1794 | 0.0659 | 0.0787 | 0.0773 | 0.1027 | 0.0953 | 0.1259 | 0.1089 | 0.1355 |
| **Glyphosate** | 0.0031 | 0.0048 | 0.0032 | 0.0042 | 0.0035 | 0.0050 | 0.0038 | 0.0053 | 0.0042 | 0.0050 |
| **Imazalil** | 0.7056 | 1.1912 | 0.6204 | 0.6731 | 0.7081 | 0.7954 | 0.7954 | 0.8462 | 0.9286 | 1.0210 |
| **Imidacloprid** | 0.0338 | 0.0408 | 0.0628 | 0.0577 | 0.0827 | 0.0737 | 0.0865 | 0.0744 | 0.1316 | 0.0819 |
| **Iprodione** | 0.1239 | 0.1798 | 0.0997 | 0.0992 | 0.1215 | 0.1435 | 0.1346 | 0.1470 | 0.1651 | 0.1887 |
| **lambda Cyhalothrin** | 0.0082 | 0.0101 | 0.0074 | 0.0062 | 0.0092 | 0.0085 | 0.0104 | 0.0091 | 0.0134 | 0.0124 |
| **Malathion** | 0.0003 | 0.0004 | 0.0003 | 0.0004 | 0.0003 | 0.0003 | 0.0003 | 0.0004 | 0.0004 | 0.0006 |
| **Methamidophos** | 0.0002 | 0.0003 | 0.0003 | 0.0003 | 0.0003 | 0.0004 | 0.0003 | 0.0004 | 0.0003 | 0.0004 |
| **Profenofos** | 0.0000 | 0.0001 | 0.0000 | 0.0000 | 0.0000 | 0.0000 | 0.0000 | 0.0000 | 0.0001 | 0.0001 |
| **Pyrethrins** | 0.0020 | 0.0019 | 0.0021 | 0.0019 | 0.0022 | 0.0020 | 0.0021 | 0.0018 | 0.0022 | 0.0020 |
| **Spinosad** | 0.1338 | 0.2193 | 0.1069 | 0.1592 | 0.1118 | 0.1439 | 0.1350 | 0.1355 | 0.2212 | 0.1620 |
| **Tebuconazole** | 0.0281 | 0.0413 | 0.0246 | 0.0245 | 0.0307 | 0.0366 | 0.0334 | 0.0390 | 0.0412 | 0.0509 |
| **Thiabendazole** | 0.2332 | 0.3622 | 0.2341 | 0.2524 | 0.2672 | 0.2747 | 0.2866 | 0.2777 | 0.3313 | 0.3228 |

NMF: Non-Negative Matrix Factorization; SD: Standard Deviation

Supplementary Table 11: Expected links between metabolites and parent compounds

| ,,,,,,,,,,,,,,,,,,METABOLITES  PESTICIDES | DMP | DMTP | DMDTP | DEP | DETP | CPO | TCP | PBA | TBZ-OH | TEB-OH |
| --- | --- | --- | --- | --- | --- | --- | --- | --- | --- | --- |
| Chlorpyrifos |  |  |  | * | * | * | * |  |  |  |
| Cypermethrin |  |  |  |  |  |  |  | * |  |  |
| Dimethoate | * | * | * |  |  |  |  |  |  |  |
| Lambda-Cyhalothrin |  |  |  |  |  |  |  | * |  |  |
| Malathion | * | * | * |  |  |  |  |  |  |  |
| Omethoate | * | * |  |  |  |  |  |  |  |  |
| Tebuconazole |  |  |  |  |  |  |  |  |  | * |
| Thiabendazole |  |  |  |  |  |  |  |  | * |  |

DMP= dimethylphosphate
DMTP= dimethylthiophosphate
DMDTP= dimethyldithiophosphate
DEP= diethylphosphate
DETP= diethylthiophosphate
CPO=chlorpyrifos-oxon
TCP=3,5,6-trichloropyridinol
PBA=3-phenoxybenzoic acid
TBZ-OH=5-hydroxy-thiabendazole
TEB-OH=hydroxy-tebuconazole

Supplementary Table 12: Urinary concentrations (µg/g creatinine) for parent compounds and metabolites in NMF Components 1 and 2 quintiles, NutriNet-Santé Study (N=296)

| **NMF Component 1** | **Quintile 1** | **N=73** | **Quintile 2** | **N=61** | **Quintile 3** | **N=45** | **Quintile 4** | **N=59** | **Quintile 5** | **N=58** |
| --- | --- | --- | --- | --- | --- | --- | --- | --- | --- | --- |
| **Variable** | **Mean** | **SD** | **Mean** | **SD** | **Mean** | **SD** | **Mean** | **SD** | **Mean** | **SD** |
| Malathion | 0.0027 | 0.0075 | **0.0057** | 0.0237 | 0.0055 | 0.0149 | 0.0036 | 0.0095 | 0.0032 | 0.0065 |
| Chlorpyrifos | 0.0119 | 0.0125 | 0.0100 | 0.0000 | **0.0231** | 0.0880 | 0.0105 | 0.0027 | 0.0108 | 0.0034 |
| CPO | 0.0025 | 0.0003 | 0.0025 | 0.0003 | **0.0027** | 0.0011 | 0.0025 | 0.0000 | 0.0025 | 0.0003 |
| Free TCP | 0.1041 | 0.0247 | **0.1331** | 0.2586 | 0.1000 | 0.0000 | 0.1122 | 0.0605 | 0.1000 | 0.0000 |
| Total TCP | 0.1734 | 0.1813 | 0.2321 | 0.5007 | 0.1778 | 0.2038 | 0.2132 | 0.2863 | **0.2722** | 0.4131 |
| Free TBZ-OH | 0.0160 | 0.0058 | **0.0202** | 0.0403 | 0.0183 | 0.0178 | 0.0168 | 0.0078 | 0.0192 | 0.0237 |
| Total TBZ-OH | 0.1091 | 0.2648 | 0.1419 | 0.3364 | 0.1237 | 0.3197 | 0.1713 | 0.3270 | **0.2035** | 0.3684 |
| Free TEB-OH | 0.0160 | 0.0058 | 0.0150 | 0.0000 | 0.0158 | 0.0052 | **0.0164** | 0.0111 | 0.0156 | 0.0046 |
| Total TEB-OH | 0.0956 | 0.1914 | 0.2618 | 0.7383 | 0.1827 | 0.2926 | **0.3480** | 0.7730 | 0.2772 | 0.4669 |
| DEP | 9.1971 | 15.9250 | 10.7730 | 26.3946 | 10.6182 | 13.6479 | 11.5425 | 23.8589 | **21.6200** | 41.6518 |
| DETP | 0.1958 | 0.2371 | 0.5605 | 1.2684 | 0.2824 | 0.4465 | 0.4700 | 1.1427 | **0.9300** | 2.0208 |
| DEDTP | **0.1000** | 0.0000 | **0.1000** | 0.0000 | **0.1000** | 0.0000 | **0.1000** | 0.0000 | **0.1000** | 0.0000 |
| DMP | 8.9886 | 14.7276 | **20.0639** | 54.2045 | 17.5842 | 52.1088 | 5.9508 | 11.2045 | 13.4643 | 24.5981 |
| DMTP | 1.3081 | 3.5770 | 3.3069 | 5.8996 | 2.8338 | 4.9669 | 3.8168 | 10.2745 | **4.7703** | 8.4817 |
| DMDTP | 0.1000 | 0.0000 | 0.1000 | 0.0000 | 0.1044 | 0.0298 | 0.1034 | 0.0260 | **0.1069** | 0.0368 |
| PBA | 0.0259 | 0.0668 | 0.0397 | 0.1135 | 0.0214 | 0.0266 | 0.0265 | 0.0402 | **0.0567** | 0.1641 |
| crMP* | 0.1450 | 0.2670 | 0.1821 | 0.4027 | 0.2129 | 0.4099 | 0.1341 | 0.2698 | **0.2393** | 0.3815 |
| crEP* | 0.1206 | 0.2521 | 0.1418 | 0.3749 | 0.1413 | 0.2245 | 0.1719 | 0.4683 | **0.2681** | 0.4762 |
| crDAP* | 0.2656 | 0.4126 | 0.3239 | 0.5895 | 0.3541 | 0.4694 | 0.3059 | 0.6069 | **0.5073** | 0.5919 |
|  |  |  |  |  |  |  |  |  |  |  |
|  |  |  |  |  |  |  |  |  |  |  |
| **NMF Component 2** | **Quintile 1** | **N=79** | **Quintile 2** | **N=54** | **Quintile 3** | **N=36** | **Quintile 4** | **N=52** | **Quintile 5** | **N=75** |
| **Variable** | **Mean** | **SD** | **Mean** | **SD** | **Mean** | **SD** | **Mean** | **SD** | **Mean** | **SD** |
| Malathion | 0.0021 | 0.0051 | 0.0015 | 0.0000 | 0.0025 | 0.0053 | **0.0083** | 0.0284 | 0.0056 | 0.0114 |
| Chlorpyrifos | **0.0194** | 0.0673 | 0.0103 | 0.0020 | 0.0100 | 0.0000 | 0.0103 | 0.0021 | 0.0104 | 0.0024 |
| CPO | **0.0026** | 0.0009 | 0.0025 | 0.0000 | 0.0025 | 0.0000 | 0.0025 | 0.0003 | 0.0025 | 0.0003 |
| Free TCP | **0.1275** | 0.2277 | 0.1000 | 0.0000 | 0.1083 | 0.0348 | 0.1029 | 0.0208 | 0.1056 | 0.0485 |
| Total TCP | 0.2357 | 0.4679 | 0.1813 | 0.1633 | 0.1694 | 0.1569 | 0.1775 | 0.1651 | **0.2593** | 0.4189 |
| Free TBZ-OH | 0.0173 | 0.0140 | 0.0156 | 0.0048 | 0.0169 | 0.0081 | 0.0157 | 0.0049 | **0.0225** | 0.0416 |
| Total TBZ-OH | **0.1659** | 0.4282 | 0.1439 | 0.2963 | 0.0868 | 0.1517 | 0.1632 | 0.3469 | 0.1548 | 0.2481 |
| Free TEB-OH | **0.0165** | 0.0103 | 0.0163 | 0.0067 | 0.0160 | 0.0058 | 0.0150 | 0.0000 | 0.0150 | 0.0000 |
| Total TEB-OH | 0.2143 | 0.5666 | **0.3431** | 0.8986 | 0.1142 | 0.1301 | 0.2056 | 0.3303 | 0.2336 | 0.4256 |
| DEP | 10.3803 | 21.3071 | 9.6883 | 17.5890 | 10.3106 | 14.9117 | 11.4021 | 16.9487 | **19.1204** | 41.5299 |
| DETP | 0.2751 | 0.5859 | 0.2733 | 0.8566 | 0.6403 | 1.2965 | 0.5448 | 1.0716 | **0.7332** | 1.8122 |
| DEDTP | **0.1000** | 0.0000 | **0.1000** | 0.0000 | **0.1000** | 0.0000 | **0.1000** | 0.0000 | **0.1000** | 0.0000 |
| DMP | 16.6227 | 42.6553 | 12.6439 | 22.5935 | 13.6897 | 40.9248 | 10.2322 | 44.7589 | 10.4336 | 19.9796 |
| DMTP | 1.5951 | 2.7772 | 1.9002 | 4.3963 | 2.9431 | 5.7832 | 2.8917 | 4.6175 | **5.8888** | 11.6385 |
| DMDTP | 0.1000 | 0.0000 | 0.1037 | 0.0272 | 0.1000 | 0.0000 | 0.1000 | 0.0000 | **0.1080** | 0.0395 |
| PBA | 0.0220 | 0.0479 | 0.0135 | 0.0131 | 0.0511 | 0.1372 | **0.0615** | 0.1802 | 0.0350 | 0.0537 |
| crMP* | 0.1719 | 0.3280 | 0.1860 | 0.2746 | 0.1242 | 0.3021 | 0.1259 | 0.3795 | **0.2457** | 0.3980 |
| crEP* | 0.1097 | 0.2481 | 0.1875 | 0.3887 | 0.1393 | 0.2500 | 0.1396 | 0.2544 | **0.2459** | 0.5523 |
| crDAP* | 0.2814 | 0.4510 | 0.3733 | 0.4874 | 0.2635 | 0.3950 | 0.2654 | 0.4442 | **0.4915** | 0.7327 |

Values in bold denote the highest value between quintiles

DMP= dimethylphosphate
DMTP= dimethylthiophosphate
DMDTP= dimethyldithiophosphate
DEP= diethylphosphate
DETP= diethylthiophosphate
CPO=chlorpyrifos-oxon
TCP=3,5,6-trichloropyridinol
PBA=3-phenoxybenzoic acid
TBZ-OH=5-hydroxy-thiabendazole
TEB-OH=hydroxy-tebuconazole

*Molar sums calculated as follows:

crMP=Total MPs= $\frac{[\mathrm{DMP}]}{125}+\frac{[\mathrm{DMTP}]}{141}+\frac{[\mathrm{DMDTP}]}{157}$

crEP=Total EPs=$\frac{[\mathrm{DEP}]}{153}+\frac{[\mathrm{DETP}]}{169}+\frac{[\mathrm{DEDTP}]}{186}$
crDAP=Total DAPs=Total MPs + Total EPs

Supplementary Table 13: Urinary concentrations (µg/g creatinine) for parent compounds and metabolites in NMF Components 3 and 4 quintiles, NutriNet-Santé Study (N=296)

| **NMF Component 3** | **Quintile 1** | **N=56** | **Quintile 2** | **N=59** | **Quintile 3** | **N=59** | **Quintile 4** | **N=65** | **Quintile 5** | **N=57** |
| --- | --- | --- | --- | --- | --- | --- | --- | --- | --- | --- |
| **Variable** | **Mean** | **SD** | **Mean** | **SD** | **Mean** | **SD** | **Mean** | **SD** | **Mean** | **SD** |
| Malathion | **0.0056** | 0.0138 | 0.0026 | 0.0054 | 0.0030 | 0.0083 | 0.0050 | 0.0227 | 0.0039 | 0.0105 |
| Chlorpyrifos | 0.0100 | 0.0000 | **0.0208** | 0.0768 | 0.0100 | 0.0000 | 0.0111 | 0.0056 | 0.0118 | 0.0132 |
| CPO | 0.0025 | 0.0000 | **0.0027** | 0.0010 | 0.0025 | 0.0000 | 0.0025 | 0.0003 | 0.0025 | 0.0003 |
| Free TCP | **0.1361** | 0.2699 | 0.1051 | 0.0274 | 0.1000 | 0.0000 | 0.1065 | 0.0521 | 0.1053 | 0.0278 |
| Total TCP | **0.2791** | 0.5273 | 0.2236 | 0.4022 | 0.1656 | 0.1497 | 0.1995 | 0.2582 | 0.2040 | 0.2490 |
| Free TBZ-OH | **0.0213** | 0.0423 | 0.0156 | 0.0046 | 0.0193 | 0.0171 | 0.0188 | 0.0224 | 0.0150 | 0.0000 |
| Total TBZ-OH | 0.1397 | 0.2586 | 0.1653 | 0.3610 | **0.1958** | 0.4443 | 0.1155 | 0.1768 | 0.1307 | 0.3190 |
| Free TEB-OH | 0.0156 | 0.0047 | **0.0164** | 0.0111 | 0.0156 | 0.0046 | 0.0161 | 0.0061 | 0.0150 | 0.0000 |
| Total TEB-OH | 0.1448 | 0.2280 | **0.2977** | 0.7937 | 0.2651 | 0.6271 | **0.2486** | 0.5595 | 0.1808 | 0.3125 |
| DEP | 10.9004 | 19.0242 | 13.3486 | 27.9205 | 13.3327 | 35.5366 | **16.6591** | 28.5257 | 8.3135 | 15.4591 |
| DETP | 0.7150 | 1.5438 | 0.7044 | 1.8657 | 0.4580 | 0.8200 | 0.3369 | 0.8414 | 0.2165 | 0.4005 |
| DEDTP | **0.1000** | 0.0000 | **0.1000** | 0.0000 | **0.1000** | 0.0000 | **0.1000** | 0.0000 | **0.1000** | 0.0000 |
| DMP | 11.1511 | 18.8008 | 16.0355 | 37.4674 | **19.7554** | 55.1173 | 11.0337 | 33.8860 | 6.1416 | 8.8119 |
| DMTP | 3.4798 | 5.8344 | **4.3351** | 10.5747 | 2.3125 | 3.2060 | 3.5240 | 8.3590 | 1.9379 | 4.4347 |
| DMDTP | 0.1000 | 0.0000 | **0.1102** | 0.0443 | 0.1000 | 0.0000 | 0.1000 | 0.0000 | 0.1035 | 0.0265 |
| PBA | **0.0561** | 0.1726 | 0.0347 | 0.0605 | 0.0174 | 0.0211 | 0.0402 | 0.1075 | 0.0228 | 0.0485 |
| crMP* | 0.2037 | 0.3741 | **0.2373** | 0.3823 | 0.1898 | 0.4262 | 0.1611 | 0.3293 | 0.1050 | 0.1345 |
| crEP* | 0.1756 | 0.3861 | **0.2280** | 0.5493 | 0.1106 | 0.2503 | 0.1733 | 0.2975 | 0.1478 | 0.3341 |
| crDAP* | 0.3793 | 0.6195 | **0.4653** | 0.6965 | 0.3003 | 0.4866 | 0.3344 | 0.4717 | 0.2527 | 0.3659 |
|  |  |  |  |  |  |  |  |  |  |  |
| **NMF Component 4** | **Quintile 1** | **N=64** | **Quintile 2** | **N=64** | **Quintile 3** | **N=54** | **Quintile 4** | **N=56** | **Quintile 5** | **N=58** |
| **Variable** | **Mean** | **SD** | **Mean** | **SD** | **Mean** | **SD** | **Mean** | **SD** | **Mean** | **SD** |
| Malathion | 0.0057 | 0.0143 | **0.0058** | 0.0234 | 0.0029 | 0.0069 | 0.0021 | 0.0042 | 0.0033 | 0.0084 |
| Chlorpyrifos | 0.0127 | 0.0136 | **0.0192** | 0.0738 | 0.0103 | 0.0020 | 0.0105 | 0.0028 | 0.0100 | 0.0000 |
| CPO | 0.0025 | 0.0003 | **0.0026** | 0.0009 | 0.0025 | 0.0000 | 0.0025 | 0.0003 | 0.0025 | 0.0003 |
| Free TCP | **0.1339** | 0.2529 | 0.1000 | 0.0000 | 0.1056 | 0.0286 | 0.1102 | 0.0593 | 0.1000 | 0.0000 |
| Total TCP | **0.2817** | 0.5176 | 0.1591 | 0.1303 | 0.1898 | 0.3053 | 0.2582 | 0.4039 | 0.1771 | 0.1649 |
| Free TBZ-OH | **0.0243** | 0.0451 | 0.0155 | 0.0044 | 0.0156 | 0.0048 | 0.0150 | 0.0000 | 0.0188 | 0.0168 |
| Total TBZ-OH | 0.1708 | 0.3507 | 0.0923 | 0.2430 | **0.1988** | 0.4059 | 0.1302 | 0.1981 | 0.1592 | 0.3717 |
| Free TEB-OH | **0.0174** | 0.0121 | 0.0150 | 0.0000 | 0.0150 | 0.0000 | 0.0156 | 0.0047 | 0.0156 | 0.0046 |
| Total TEB-OH | 0.2587 | 0.5661 | 0.0814 | 0.1162 | **0.3595** | 0.9258 | 0.2903 | 0.5021 | 0.1784 | 0.3124 |
| DEP | 10.3767 | 23.5812 | 12.1106 | 21.7827 | 11.6113 | 19.9084 | **16.7680** | 39.4792 | 12.6917 | 24.1186 |
| DETP | 0.2897 | 0.5581 | 0.3678 | 0.9653 | **0.9794** | 2.2267 | 0.3343 | 0.5834 | 0.5029 | 1.0261 |
| DEDTP | **0.1000** | 0.0000 | **0.1000** | 0.0000 | **0.1000** | 0.0000 | **0.1000** | 0.0000 | **0.1000** | 0.0000 |
| DMP | **16.6611** | 38.4331 | 14.1950 | 34.2897 | 15.9509 | 39.3578 | 5.5813 | 7.9738 | 11.2878 | 42.6857 |
| DMTP | 2.2975 | 3.9331 | 2.9063 | 6.5259 | **4.1131** | 8.4438 | 3.1705 | 9.5051 | 3.3431 | 6.2723 |
| DMDTP | 0.1031 | 0.0250 | 0.1000 | 0.0000 | **0.1037** | 0.0272 | 0.1036 | 0.0267 | 0.1034 | 0.0263 |
| PBA | 0.0412 | 0.1531 | **0.0420** | 0.1245 | 0.0350 | 0.0517 | 0.0227 | 0.0367 | 0.0284 | 0.0451 |
| crMP* | 0.1987 | 0.3212 | **0.2446** | 0.4680 | 0.1737 | 0.2672 | 0.0982 | 0.1192 | 0.1693 | 0.4125 |
| crEP* | 0.1493 | 0.4720 | 0.1935 | 0.4065 | 0.1434 | 0.2694 | **0.1972** | 0.4270 | 0.1513 | 0.2372 |
| crDAP* | 0.3479 | 0.6217 | **0.4380** | 0.6731 | 0.3170 | 0.3880 | 0.2953 | 0.4681 | 0.3205 | 0.4693 |

DMP= dimethylphosphate
DMTP= dimethylthiophosphate
DMDTP= dimethyldithiophosphate
DEP= diethylphosphate
DETP= diethylthiophosphate
CPO=chlorpyrifos-oxon
TCP=3,5,6-trichloropyridinol
PBA=3-phenoxybenzoic acid
TBZ-OH=5-hydroxy-thiabendazole
TEB-OH=hydroxy-tebuconazole

*Molar sums calculated as follows:

crMP=Total MPs= $\frac{[\mathrm{DMP}]}{125}+\frac{[\mathrm{DMTP}]}{141}+\frac{[\mathrm{DMDTP}]}{157}$

crEP=Total EPs=$\frac{[\mathrm{DEP}]}{153}+\frac{[\mathrm{DETP}]}{169}+\frac{[\mathrm{DEDTP}]}{186}$
crDAP=Total DAPs=Total MPs + Total EPs
